# Beyond Earth: Recent Advancements in Microgravity Biomedical and Genetic Research in Saudi Arabia

**DOI:** 10.3390/ijms27177613

**Published:** 2026-08-25

**Authors:** Yousef M. Hawsawi, Yahya F. Jamous, Shouq F. Alghannam, Hala Aldahshan, Loulwah Alothman, Rawan Fitaihi, Nouf Aljawini, Sana S. Alqarni, Rihaf Alfaraj, Esraa A. Aldkheil, Sarah S. Alotaibi

**Affiliations:** 1 Research Center, King Faisal Specialist Hospital and Research Centre, P.O. Box 40047, Jeddah 21499, Saudi Arabia; 2 Department of Biochemistry and Molecular Medicine, College of Medicine, Alfaisal University, P.O. Box 50927, Riyadh 11533, Saudi Arabia; 3 Wellness and Preventive Medicine Institute, Health Sector, King Abdulaziz City for Science and Technology (KACST), Riyadh 11442, Saudi Arabia; yjamous@kacst.gov.sa (Y.F.J.); salghannam@kacst.gov.sa (S.F.A.); 4 Department of Clinical Laboratory Sciences, College of Applied Medical Sciences, King Saud University, Riyadh 12372, Saudi Arabia; haldahshan@ksu.edu.sa (H.A.); saalqarni@ksu.edu.sa (S.S.A.); 5 Department of Oral Medicine and Diagnostic Sciences, College of Dentistry, King Saud University, Riyadh 12371, Saudi Arabia; loalothman@ksu.edu.sa; 6 Department of Pharmaceutics, College of Pharmacy, King Saud University, Riyadh 11451, Saudi Arabia; rfitaihi@ksu.edu.sa (R.F.); ralfaraj@ksu.edu.sa (R.A.); 7 Department of Community Health Sciences, College of Applied Medical Sciences, King Saud University, Riyadh 11433, Saudi Arabia; naljawini@ksu.edu.sa; 8 Bioengineering Institute, Health Sector, King Abdulaziz City for Science and Technology (KACST), Riyadh 11442, Saudi Arabia; ealdkheil@kacst.gov.sa; 9 Applied Genomic Technologies Institute, King Abdulaziz City for Science and Technology (KACST), Riyadh 11442, Saudi Arabia

**Keywords:** microgravity, space medicine, genomics, multi-omics, precision medicine, space biology, artificial intelligence, Saudi Arabia

## Abstract

Microgravity research has emerged as a rapidly evolving field at the intersection of space medicine, genomics, biotechnology, and precision medicine. Exposure to the space environment induces complex physiological and molecular adaptations that affect multiple biological systems, including immune regulation, metabolism, musculoskeletal function, and gene expression. Recent advances in genomics, multi-omics technologies, artificial intelligence, and bioengineering have substantially improved our understanding of biological adaptation to spaceflight and expanded opportunities for translational biomedical research. This review summarizes recent advances in genetic and biomedical research under microgravity conditions, with particular emphasis on molecular mechanisms, omics technologies, genome editing, microbiome research, regenerative medicine, and personalized healthcare approaches. Major experimental platforms, landmark spaceflight studies, and translational applications in infectious diseases, cancer biology, aging, tissue engineering, and pharmaceutical development are discussed. The review also highlights Saudi Arabia’s emerging contributions to genomic medicine and space biosciences through initiatives such as the Saudi Human Genome Program, the Saudi Pangenome Project, the Saudi Space Agency, and the BioGravity Initiative. Recent Saudi participation in human spaceflight and microgravity-associated biomedical research is discussed within the context of Vision 2030 and national investments in biotechnology and precision medicine. Collectively, advances in microgravity research are expected to contribute to the advancement of precision medicine and facilitate the development of innovative diagnostic and therapeutic strategies with significant implications for both human space exploration and terrestrial healthcare.

## 1. Introduction

Space exploration has reached a transformative phase in which human missions are moving beyond short-term orbital activity toward prolonged habitation in low Earth orbit, lunar exploration, and future deep-space missions. This shift has intensified the need to understand how the human body actively responds to the combined stressors of microgravity, cosmic radiation, confinement, altered circadian rhythms, and environmental isolation [1,2]. Spaceflight is recognized not only as an engineering challenge but also as a complex biomedical environment that induces systemic physiological and molecular adaptations across multiple organs and biological pathways [1,2,3,4]. These adaptations are particularly relevant during long-duration missions, where even subtle biological changes can accumulate and affect astronaut health, performance, and post-mission recovery [2,3].

Microgravity represents one of the most distinctive environmental stressors encountered during spaceflight. Unlike terrestrial gravity, microgravity reduces mechanical loading and alters fluid distribution, cellular mechanotransduction, cytoskeletal organization, and tissue-level homeostasis [1,4,5]. Human and experimental studies have demonstrated that spaceflight-associated physiological changes may involve the musculoskeletal, cardiovascular, immune, reproductive, and nervous systems [2,6,7,8]. Cardiovascular remodeling, endothelial dysfunction, immune dysregulation, bone loss, muscle atrophy, and altered reproductive biology have all been reported as important biomedical concerns in space medicine [6,7,8]. Therefore, microgravity research is important for identifying health risks, developing countermeasures, and improving the safety of future human space exploration.

At the molecular level, spaceflight and simulated microgravity are associated with alterations in gene expression, mitochondrial function, oxidative stress pathways, DNA damage responses, immune signaling, and epigenetic regulation [4,9,10,11].

Multi-omics studies suggest that mitochondrial stress may act as a major biological mediator of spaceflight adaptation, linking energy metabolism, oxidative stress, immune regulation, and tissue adaptation [4]. In addition, studies of microgravity-associated gene regulation have shown that gravitational unloading may affect transcriptional networks, non-coding RNA regulation, DNA methylation, and cellular stress responses across various cell types and organisms [9]. Collectively, these findings indicate that microgravity can influence cellular function across genomic, transcriptomic, epigenomic, proteomic, and metabolic levels.

The growing application of omics technologies has expanded the molecular characterization of biological responses to spaceflight. Subsequent multi-omics investigations across human, animal, and cellular datasets further supported the idea that spaceflight produces coordinated molecular signatures involving mitochondrial dysregulation, immune modulation, oxidative stress, and DNA repair pathways [4]. More recently, large-scale space omics platforms, including NASA GeneLab and the Space Omics and Medical Atlas, have expanded access to integrated genomic, transcriptomic, proteomic, metabolomic, microbiome, and clinical datasets, thereby enabling deeper investigation of biological adaptation to spaceflight [5,12].

The integration of genomics with space biology has improved our understanding of biological adaptation to extreme environments. Historically, studies of spaceflight-associated health effects relied primarily on physiological findings such as muscle wasting, cardiovascular deconditioning, and bone loss. Nevertheless, the development of high-throughput sequencing technologies has shifted research efforts toward identifying the molecular mechanisms underlying these phenotypic changes [13,14]. Contemporary research indicates that microgravity affects not merely individual genes but also complex regulatory networks involving transcription factors, epigenetic modifications, cellular signaling pathways, and inter-organ communication systems [13,15]. Therefore, genomic and systems biology approaches have become important tools for characterizing biological responses to spaceflight and informing the development of precision countermeasures for future missions.

Recent multi-omics studies have demonstrated that microgravity-induced molecular adaptations occur through several biological levels, such as the genome, transcriptome, proteome, metabolome, and microbiome [16,17]. Studies conducted under both real and simulated microgravity conditions have identified alterations in mechanisms associated with cellular metabolism, immune regulation, mitochondrial activity, oxidative stress responses, DNA repair, and tissue regeneration [18,19]. Several of these molecular signatures appear to be evolutionarily conserved across species, suggesting the existence of fundamental biological adaptation pathways triggered by gravitational changes [16]. These findings support the use of microgravity studies as an experimental platform for investigating biological pathways relevant to both astronaut health and terrestrial medicine.

The emergence of omics technologies has also expanded opportunities for translational research and precision medicine. Large-scale datasets generated from astronauts, animal models, and cellular systems have enabled the identification of candidate biomarkers related to physiological resilience, susceptibility to environmental stressors, and disease progression [17,20]. Moreover, advances in artificial intelligence, bioinformatics, and systems biology have enabled the combination of multidimensional datasets, allowing researchers to model complex biological responses to spaceflight with increased accuracy [21]. These advances may support predictive and personalized approaches to astronaut healthcare and could also inform broader precision medicine strategies on Earth.

Beyond human health, microgravity-based genomic research has generated substantial interest in tissue engineering, regenerative medicine, and biotechnology. Exposure to microgravity has been shown to facilitate the formation of three-dimensional cellular structures, organoids, and multicellular spheroids that more closely resemble native tissues than traditional two-dimensional culture systems [22,23]. These models have become valuable platforms for investigating cancer biology, stem cell behavior, tissue regeneration, drug screening, and disease modeling. Thus, microgravity research provides a distinctive experimental platform for biomedical investigation and for understanding human adaptation to the space environment.

Despite substantial development of space biology, genomics, and biomedical engineering, several important knowledge gaps remain. Although numerous studies have characterized the physiological and molecular influences of microgravity, several underlying mechanisms governing long-term adaptation, disease susceptibility, and inter-individual differences remain incompletely understood [24,25]. Moreover, numerous studies have relied on astronauts from a limited number of international missions, while the combination of large-scale genomic datasets, artificial intelligence-driven analyses, and precision medicine frameworks remains in its early stages [20,21]. Addressing these limitations will be important for developing evidence-based countermeasures for future long-duration missions to the Moon, Mars, and other deep-space destinations.

In parallel, the convergence of space science, biotechnology, genomics, and digital health technologies provides opportunities for scientific advancement, international collaboration, and innovation. Advances in multi-omics integration, machine learning, organoid technologies, and tissue engineering may improve the study of human adaptation to extreme environments and support healthcare applications for astronauts and terrestrial populations [16,21,22]. Findings from microgravity research may have translational relevance to age-related diseases, cancer biology, immune disorders, cardiovascular dysfunction, regenerative medicine, and pharmaceutical development on Earth [17,19,23].

Saudi Arabia is increasingly positioned to contribute to this rapidly evolving scientific landscape. Through Vision 2030, the Saudi Human Genome Program, investments in biotechnology and artificial intelligence, and the development of national research infrastructure, the Kingdom has expanded its capacity for biomedical and genomic research [24,25]. Furthermore, recent initiatives established by the Saudi Space Agency, integrated with increasing participation in international space missions and space-health collaborations, have created new opportunities to combine genomic sciences, precision medicine, and space biosciences within a unified research framework [26,27]. These developments are aligned with global efforts to advance personalized healthcare, biomedical innovation, and human space exploration.

Overall, a comprehensive review of recent advances in microgravity-related biomedical and genetic research is timely. This review outlines the current understanding of the biological and molecular effects of microgravity, recent advances in technologies such as omics and bioengineering approaches, major experimental platforms, and their potential biomedical applications. In addition, it investigates Saudi Arabia’s advances in genomic and space bioscience studies. By integrating findings from space biology, genomics, biotechnology, and translational medicine, this review summarizes major advances, identifies current limitations, and highlights future research directions. Furthermore, it considers the increasing contribution of Saudi Arabia in supporting innovation in genomics, space medicine, and biomedical research.

## 2. Genetic and Microgravity Research in Saudi Arabia

**Figure 1:**
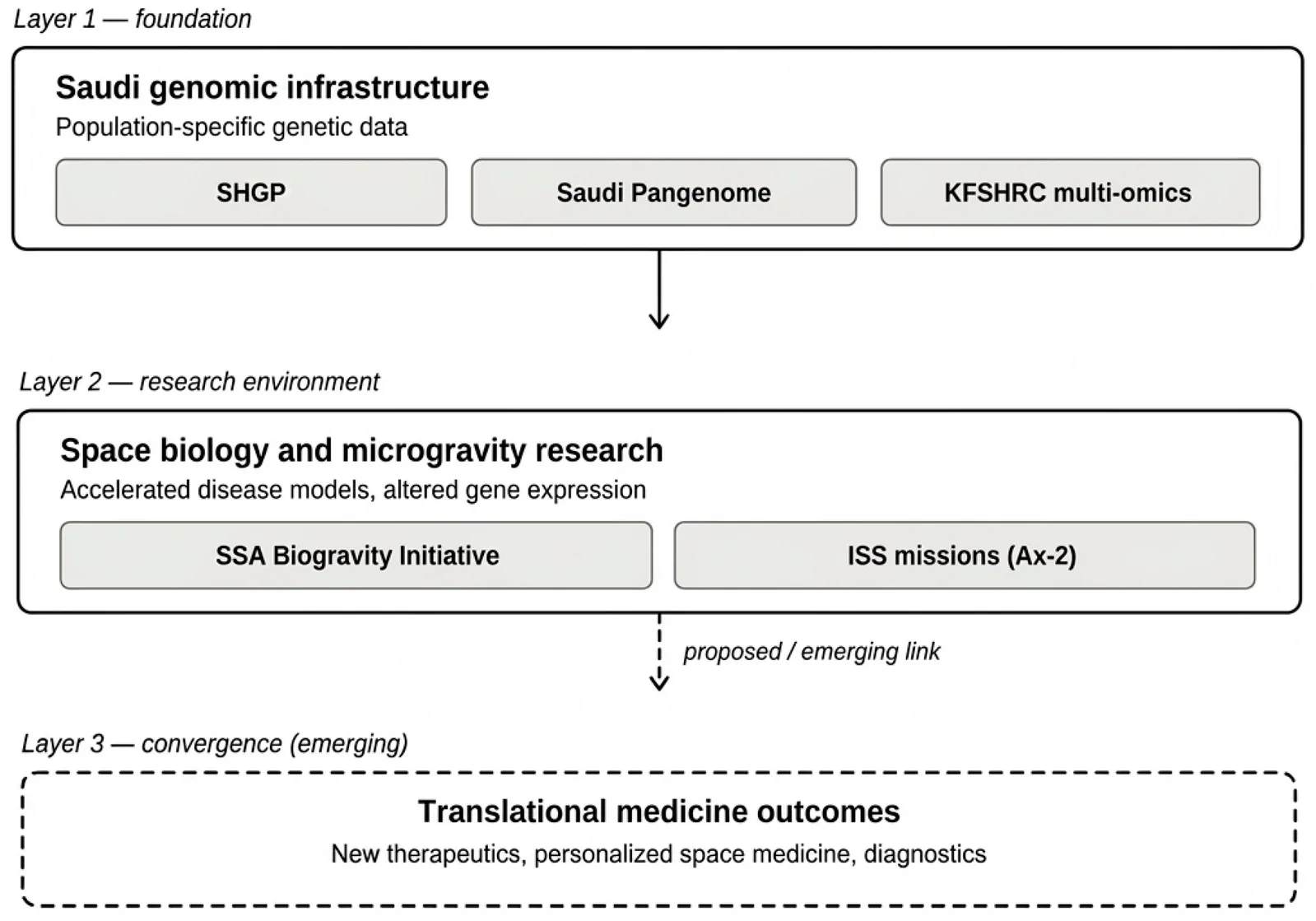
Conceptual framework Saudi genomic programs × space biology × translational medicine. The foundational layer is the Saudi genomic infrastructure (the SHGP, the Saudi Pangenome, and KFSHRC multi-omics), which provides population-specific genetic data. Space biology and microgravity research, conducted through the SSA’s Biogravity Initiative and ISS missions such as Ax-2, draw on this infrastructure to study accelerated disease models and altered gene expression. The two domains converge on translational medicine outcomes: new therapeutics, personalized space medicine, and diagnostics for both astronauts and terrestrial patients. Figure created by using FigureLabs during an active paid subscription, with authorization for academic publication.

Over the past decade, Saudi Arabia has invested in two distinct biomedical fields: genomic medicine and space biology [25,28]. Although these fields initially developed separately, recent Saudi spaceflight research has increasingly linked genomic expertise and resources to space biology [29,30]. Genomic research provides the population-specific reference data needed to interpret molecular findings, while microgravity offers an experimental environment in which biological systems can be observed under sustained physiological stress. Together, these fields create a framework for studying spaceflight responses in a Saudi context (Figure 1).

The genomic infrastructure emerged in response to a domestic clinical burden. Elevated rates of consanguinity in Saudi Arabia are associated with a high prevalence of rare autosomal recessive disorders [31,32], which created an early demand for large-scale diagnostic sequencing and population-based screening. More recently, this sequencing and analytical capacity has also become relevant to spaceflight-related research. In this context, the Biogravity Initiative conducted during Axiom Mission 2 (Ax-2) drew on Saudi expertise in genomics and biomedical data analysis, largely developed through clinical research programs [29,33]. Interpretation of microgravity-induced molecular changes depends on a well-characterized genomic reference against which such changes can be read [34].

The Saudi Human Genome Program (SHGP) and the Saudi Pangenome supply such a reference for the Saudi population [24,35]. Together, these population-matched datasets can help distinguish baseline genetic variation from individual molecular responses to spaceflight. These resources could eventually inform more personalized approaches to space medicine [36].

Despite substantial progress, the field remains at an early stage of development. Although the infrastructure and funding mechanisms are largely in place, few peer-reviewed studies have directly linked Saudi genomic research to space biology. This emerging work is recent, preliminary, and largely exploratory, and its long-term clinical and translational implications remain to be established.

### Historical Development of Genetic and Microgravity Research

Genetic research in Saudi Arabia initially developed within a clinical context. In the late 20th century, the focus was on inherited metabolic and neurogenetic disorders [37]. High consanguinity meant a heavy burden of recessive disease, which raised demand for accurate diagnosis and early screening. The King Faisal Specialist Hospital and Research Centre (KFSHRC) conducted foundational work in early clinical genetics, newborn screening, and the patient datasets that later national sequencing projects drew on [37]. At the national level, the King Abdulaziz City for Science and Technology (KACST) coordinated funding and broader research priorities [38]. Over time, this evolved into a division of labor: hospitals generate clinical and phenotypic data, whereas KACST handles national research planning and infrastructure.

The SHGP, launched in 2013, set out to sequence 100,000 genomes and characterize population-specific genetic variation in Saudi Arabia [39]. It let researchers identify thousands of pathogenic variants, many of which were built into targeted screening programs to reduce the burden of severe inherited disease [27,39]. The SHGP began as a large-scale discovery initiative, with its most immediate impact observed in disease prevention and early detection (Table 1).

Two recent developments have improved Saudi genomic reference datasets. KSA001, the first Telomere-to-Telomere (T2T) Saudi genome, closed gaps in earlier reference assemblies. The Saudi Pangenome project went further, representing regional genetic diversity rather than relying on a single reference genome [35].

Saudi Arabia’s involvement in space research began in 1985, when Prince Sultan bin Salman Al Saud took part in a US space shuttle mission. A more structured national program came decades later with the Saudi Space Agency (SSA), which formalized and expanded the country’s space activities [40]. In 2023, the Ax-2 mission sent Rayyanah Barnawi and Ali AlQarni to the International Space Station (ISS) [29].

The SSA then launched the Biogravity Initiative to support and coordinate space biology research [33]. It gives Saudi researchers a structured platform for biological experiments under microgravity. The program is new and the number of published studies is small, so its long-term sustainability and scientific impact cannot yet be assessed.

## 3. Current Trends and Technologies in Genetic and Microgravity Research

Recent progress in genetics, space biology, and computational sciences has reshaped the study of biological responses to microgravity. This field is no longer limited to observing physiological changes in astronauts; it now examines the molecular, cellular, and tissue-level mechanisms that explain adaptation to the space environment. Microgravity, ionizing radiation, altered fluid dynamics, isolation, circadian disruption, and reduced mechanical loading can influence DNA stability, gene expression, protein function, metabolism, epigenetic regulation, immune activity, and cellular organization [1,3]. The major omics and bioengineering technologies currently supporting this field are summarized in Table 2, which outlines their main functions and applications in microgravity research.

One of the main themes of modern genetic research is the use of genome-engineering technologies, especially CRISPR-Cas systems. In functional genomics, CRISPR-Cas9 has become an important tool because it can perform targeted modifications such as insertions, deletions, and sequence substitutions in DNA [41]. Prime editing is one of the newer approaches that has enhanced editing accuracy by allowing targeted correction without double-strand DNA breaks or donor DNA templates [42].

Additionally, single-cell sequencing has grown in importance in space bioscience. Single-cell RNA sequencing, in contrast to bulk sequencing, records gene expression patterns in individual cells, enabling the identification of rare subpopulations and cellular heterogeneity [43]. This is especially crucial for research involving stem cells, immune cells, organoids, and engineered tissues, since different cell types may respond differently to the same microgravity exposure, as summarized in Table 2.

Artificial intelligence is increasingly important for research in genetics and microgravity. Machine-learning models can analyze large genomic data sets, aid in experimental design, improve the design of CRISPR guide RNA and predict off-target effects [44,45]. Deep-learning tools, such as AlphaFold, have also improved predictions of protein structure and can help scientists better understand protein function and molecular interactions under stress [46].

Microgravity provides a unique experimental environment for studying gene regulation, stem-cell behavior, and tissue organization. Previous studies have shown that microgravity can alter stem-cell proliferation, differentiation, osteogenesis, hematopoiesis, and regenerative pathways [47].

**Figure 2:**
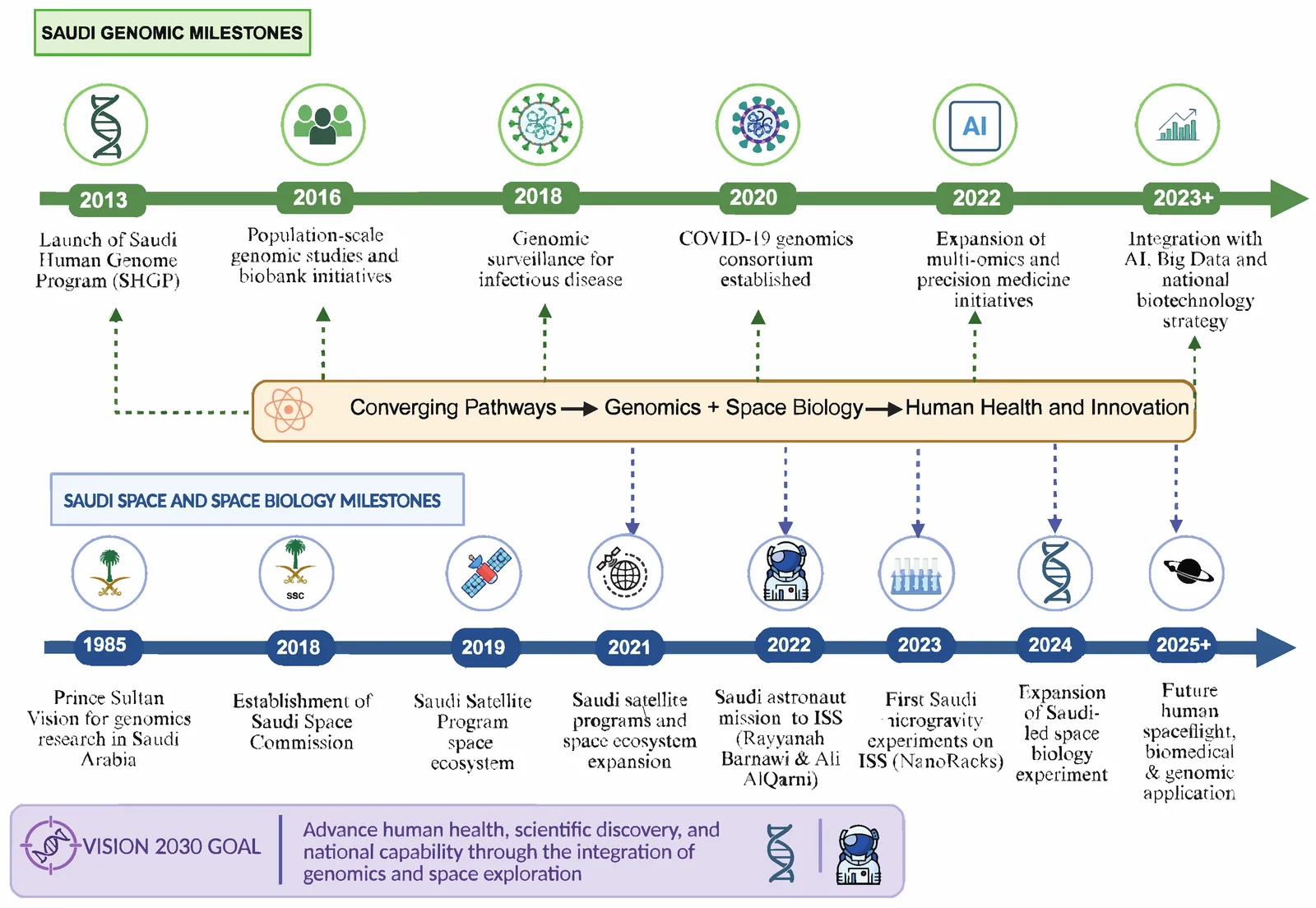
Saudi Space and Genomic Timeline: Convergence of Genomics and Space Exploration. This timeline illustrates selected Saudi milestones in genomics and space biology, including the launch of the Saudi Human Genome Program in 2013, space exploration initiatives in Saudi Arabia, highlighting key milestones from 1985 to the establishment of the Saudi Space Commission in 2018, the Saudi astronaut mission to the International Space Station in 2023, and the launch of the Saudi BioGravity initiative in 2024 [31,51,52,53]. The figure also illustrates how genomics, multi-omics, biotechnology, artificial intelligence, human spaceflight, and microgravity research are increasingly being integrated as convergent paths toward precision medicine, biomedical innovation, and future space medicine applications [25,54]. Created in BioRender. Dey, S. (2026) https://BioRender.com/koktnng (accessed on 27 June 2026).

Technological platforms have been essential for developing this field. In real microgravity, the International Space Station is the main laboratory for examining biology [48]. At the same time, several technologies such as simulated microgravity systems, portable sequencing devices, automated laboratories, live-cell imaging, organ-on-chip models, lab-on-a-chip platforms, and 3D bioprinting are increasing experimental capacity [16,49]. These technologies allow more controlled and physiologically relevant studies of cells and tissues under space-like conditions. Figure 2 illustrates the convergence of Saudi space initiatives, genomics, biotechnology, and biomedical innovation, demonstrating the broader national significance of these developments.

Overall, integrated, systems-level biology is becoming more prevalent in genetic and microgravity research. Genome editing, single-cell analysis, multi-omics profiling, bioengineering platforms, automation, and AI-driven analytics are now all included in this field. This strategy is critical for identifying microgravity-responsive pathways, developing countermeasures for astronaut health, improving tissue-engineering strategies, and translating space-based discoveries into biomedical applications on Earth [16,49,50].

**Table 2:** Emerging Omics and Bioengineering Technologies Driving Space Bioscience Research. These integrated approaches enable multi-level analysis of biological systems under microgravity conditions, supporting advancements in precision medicine, regenerative biology, and astronaut health [9,16,17,50].

Technology Category	Technology	Core Function	Application in Microgravity Research
Genomics	Whole Genome Sequencing (WGS)	Comprehensive DNA analysis	Identifies mutations and genomic instability in space-exposed cells
Transcriptomics	RNA-seq	Measures gene expression changes	Detects altered pathways under microgravity conditions
Proteomics	Mass spectrometry	Protein profiling and signaling analysis	Links gene expression to protein function in microgravity
Metabolomics	LC-MS/GC-MS	Small-molecule metabolic profiling	Reveals metabolic stress and adaptation mechanisms
Epigenomics	DNA methylation/histone modification	Gene regulation without sequence change	Identifies long-term regulatory effects of microgravity
Single-cell Omics	scRNA-seq/multi-omics	High-resolution cellular analysis	Detects cellular heterogeneity and adaptive responses
Microbiomics	16S rRNA/metagenomics	Microbial community analysis	Studies microbiome shifts affecting metabolism and immunity
Genome Editing	CRISPR-Cas systems	Targeted DNA modification	Functional validation of genes affected by microgravity
Bioengineering	3D Bioprinting	Tissue fabrication in microgravity	Enables formation of 3D tissues/organoids
Organ-on-Chip	Microfluidic systems	Simulated organ environments	Models’ human physiology under microgravity
Lab-on-a-Chip	Miniaturized platforms	Portable experimental systems	Enables autonomous experiments in space
Imaging Technologies	Live-cell imaging	Real-time cellular visualization	Tracks structural and functional changes in microgravity
Artificial Intelligence	Machine learning	Multi-omics data integration	Predicts biological responses and therapeutic targets
Automation Systems	Robotic lab systems	Automated experimentation	Enables high-throughput ISS experiments

### Space Missions and Microgravity Experiments

**Figure 3:**
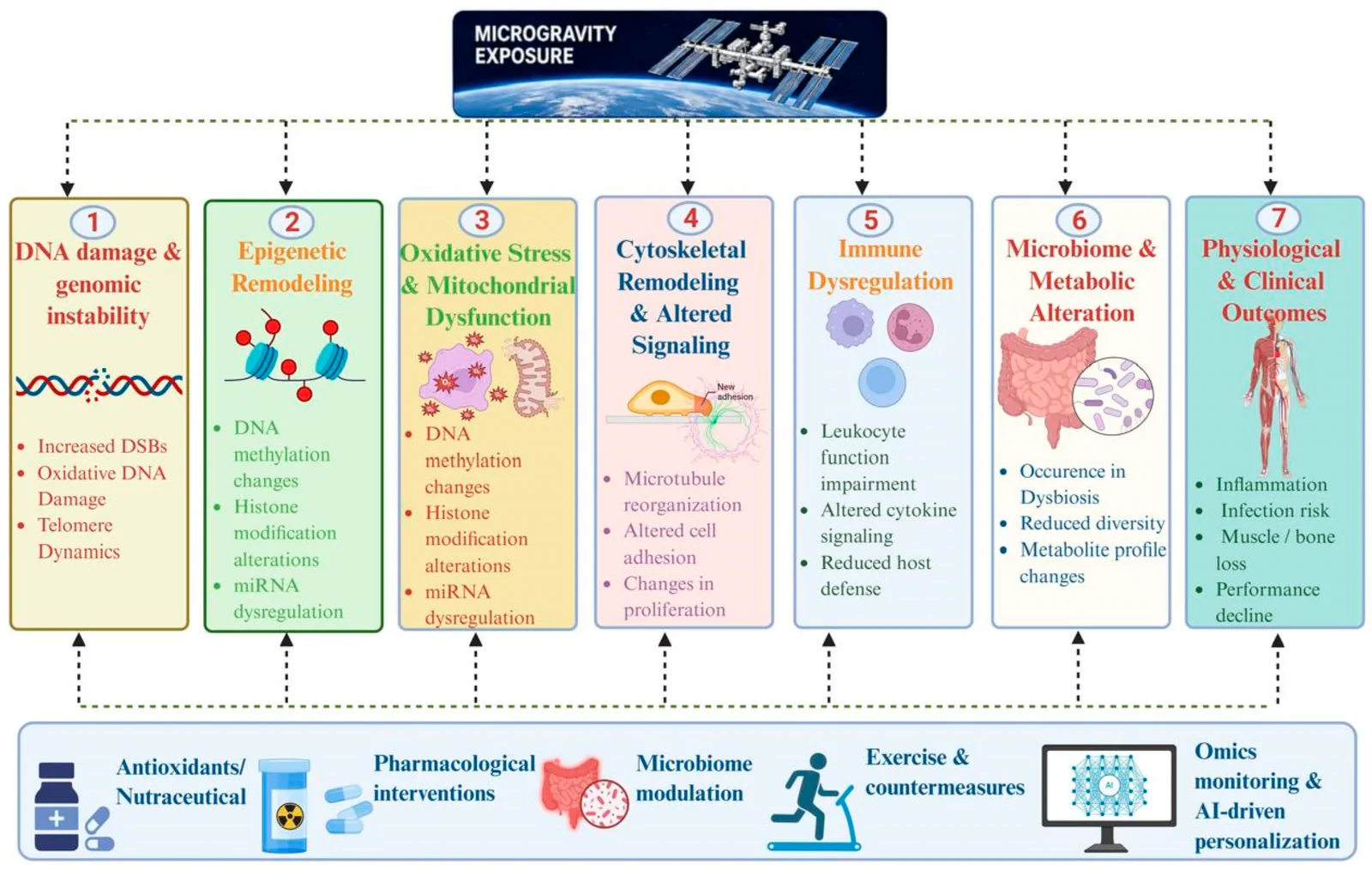
Molecular Effects of Microgravity on Biological Systems. Microgravity exposure induces a cascade of interconnected molecular and physiological responses, including DNA damage and genomic instability, epigenetic remodeling, oxidative stress and mitochondrial dysfunction, cytoskeletal remodeling, immune dysregulation, microbiome and metabolic alteration, and downstream clinical outcomes. The figure also summarizes major adaptation and countermeasure strategies, including antioxidants, pharmacological interventions, microbiome modulation, exercise-based countermeasures, and omics monitoring with AI-driven personalization [1,3,4,5]. Created in BioRender. Dey, S. (2026) https://BioRender.com/d2q0pgn (accessed on 27 June 2026).

Examining how living systems react to environmental stressors that cannot be fully replicated on Earth is made possible by the unique research environment that space missions offer. While orbital flight is characterised by microgravity, a number of interconnected variables, such as ionising radiation, altered fluid distribution, confinement, circadian disruption, operational stress, and decreased mechanical loading, influence biological responses in space. Several physiological systems are influenced by these variables, including the immune, musculoskeletal, cardiovascular, metabolic, and neuroendocrine systems. They might be involved in oxidative stress, DNA damage responses, altered mitochondria, inflammatory signalling, and modifications to gene regulation at the molecular level [1]. Figure 3 illustrates the fundamental molecular and physiological effects related to microgravity exposure.

The NASA—Twins Study, a significant contribution to human space biology, compared astronaut Scott Kelly with his identical twin Mark Kelly, who stayed on Earth, both during and after a year-long mission aboard the International Space Station. This study’s longitudinal design and integration of molecular, physiological, cognitive, immunological, and microbiome data are what make it valuable. The findings demonstrated that while some changes, such as immune activation, inflammatory markers, DNA damage responses, telomere-related changes, and transcriptional alterations, persisted for varying lengths of time, many spaceflight-associated changes returned toward baseline upon return to Earth [3]. This study highlighted the importance of repeated sampling before, during, and after spaceflight for astronaut health assessment, as opposed to a single-point assessment.

Understanding of cellular behaviour has also increased as a result of microgravity experiments. Microgravity has been demonstrated to modify cytoskeletal organization, cell adhesion, proliferation, apoptosis, differentiation, extracellular matrix remodelling, and intracellular signalling in cultured cells, stem cells, organoids, tissues, and animal models [55]. These effects are significant because a number of terrestrial disease processes, such as ageing, cancer progression, immunological dysfunction, bone loss, muscle atrophy, and tissue degeneration, are mediated by the same biological pathways. The response to microgravity is more easily understood as an interconnected network than as a linear series of discrete events, as illustrated in Figure 3.

Recently, space biology has benefited from coordinated data resources, which allow findings from various missions to be compared more systematically. One significant example is the Space Omics and Medical Atlas (SOMA), which compiles clinical, cellular, and molecular datasets from commercial and governmental missions, such as NASA, JAXA, Inspiration4, Axiom, and Polaris-related research [5]. These resources may support the development of evidence-based countermeasures and aid in the identification of recurrent biological signatures of spaceflight. Additionally, they signify a shift in precision medicine from small, mission-specific observations to a more comprehensive framework.

Saudi Arabia’s involvement in microgravity research has given this field a significant regional dimension. Saudi astronauts Rayyanah Barnawi and Ali AlQarni took part in scientific research on the International Space Station during Axiom Mission 2, including studies on human health and biological reactions in microgravity [36,53,56]. This mission was important as a human spaceflight milestone as well as a venue for international cooperation, education, and scientific engagement. Major milestones in genomic and biomedical research under microgravity, such as early biological spaceflight studies, astronaut health monitoring, multi-mission data integration, commercial space research, and Saudi contributions, are presented in Table 3, which summarises these developments.

Overall, space missions and microgravity experiments are critical for understanding how humans adapt to extreme environments. They also offer models for researching mechanisms related to ageing, inflammation, immune regulation, cancer biology, musculoskeletal degeneration, regenerative medicine, and precision medicine. However, larger astronaut cohorts, standardised procedures, long-term biological sampling, global data exchange, and a closer connection between experimental results and the creation of clinical countermeasures are all necessary for future advancements.

## 4. Applications of Genetic and Microgravity Research in Various Fields

Genetic technologies and microgravity research have applications beyond astronaut health, including infectious disease, immunology, food security, environmental biotechnology, and therapeutic development [57,58]. Studies examining biological responses to altered gravity at molecular, microbial, and physiological levels have also contributed to research in biomedical innovation, agriculture, resource management, and life-support engineering [57]. Within Saudi Arabia, emerging national initiatives in genomics and biomedical microgravity research include the Saudi Human Genome Program, the Genomic Medicine Center of Excellence at King Faisal Specialist Hospital and Research Centre, and the BioGravity initiative [39,59,60]. Together, these initiatives reflect growing national investment in genomics and biomedical microgravity research.

### 4.1. Biomedical and Healthcare Applications

Spaceflight offers a unique environment for investigating host–pathogen interactions, immune function, and the development of biomedical countermeasures. Experimental studies conducted under both orbital and simulated microgravity conditions have reported alterations in microbial physiology, including changes in virulence, stress adaptation, and antimicrobial susceptibility, although the magnitude of these effects varies among species and experimental systems. Fungal and bacterial organisms exposed to orbital or simulated microgravity frequently display enhanced pathogenic phenotypes, altered stress-response pathways, and increased resistance to environmental challenges. Notably, Aspergillus fumigatus isolates recovered from the International Space Station exhibited increased lethality compared with terrestrial controls, associated with elevated production of the mycotoxin trypacidin, while Serratia marcescens demonstrated increased virulence in Drosophila melanogaster infection models under both orbital and simulated microgravity conditions [61]. Among bacterial pathogens, Salmonella enterica serovar Typhimurium cultured under modeled microgravity exhibits altered virulence-associated gene expression, increased acid stress resistance, enhanced macrophage survival, and greater recovery from murine spleen and liver tissues compared with normal-gravity controls [62]. Subsequent orbital co-culture experiments with human intestinal epithelial cells confirmed that these changes extend to altered host–pathogen interaction dynamics at the cellular level, indicating that microgravity simultaneously reshapes both microbial behavior and host cellular response pathways [63].

Microgravity-associated changes have also been linked to altered antimicrobial susceptibility patterns, including broad-spectrum resistance phenotypes, with a substantial proportion of isolates resistant to at least one commonly used antimicrobial agent deployed aboard the station [64]. Mechanistically, many of these changes appear linked to low-fluid-shear stress environments that mimic micromechanical conditions encountered by microbes within host tissues, altering quorum sensing, biofilm architecture, membrane transport systems, and virulence-associated signaling pathways [65]. Collectively, these studies indicate that microgravity influences microbial virulence and host–pathogen interactions. However, most data are derived from experimental models, and the relevance of these findings to clinical infection risk requires further investigation [58,65].

In addition to its effects on microbial adaptation, spaceflight has been associated with alterations in both innate and adaptive immune responses, including reduced leukocyte proliferation, impaired natural killer cell cytotoxicity, altered T-cell responsiveness, and dysregulated cytokine production [66]. Even short-duration spaceflight has been associated with reduced innate immune activity and altered antioxidant gene expression. These changes may contribute to increased susceptibility to infection, although their clinical significance remains under investigation [67]. Epigenetic changes may also contribute to immune dysregulation during spaceflight. The NASA Twins Study reported alterations in DNA methylation, immune signaling, mitochondrial metabolism, inflammatory pathways, and stress-response networks during prolonged orbital exposure [3]. Reactivation of latent herpesviruses, including Epstein–Barr virus, varicella-zoster virus, herpes simplex virus-1, and cytomegalovirus, has been reported during and after spaceflight and is widely considered a marker of altered immune regulation in astronauts [68]. Nutritional interventions, including vitamin D supplementation, have been proposed as potentially modifiable factors influencing viral reactivation risk and broader immune resilience [69].

Recent developments in Saudi Arabia provide opportunities to expand research in this area. During Axiom Mission 2, Saudi astronauts participated in biomedical experiments examining immune-cell inflammatory responses under microgravity, including studies of mRNA stability and pro-inflammatory signaling in human leukocytes [70,71]. In parallel, the expansion of genomic infrastructure through the Genomic Medicine Center of Excellence at King Faisal Specialist Hospital and Research Centre has strengthened national capabilities in clinically integrated genomics, AI-assisted variant interpretation, multi-omics analysis, and biobanking [59]. The expansion of genomic and biobanking infrastructure in Saudi Arabia provides resources that could facilitate future studies in space biology and precision medicine.

### 4.2. Host–Microbiome–Immune Axis in Spaceflight

The human microbiome plays an important role in maintaining immune homeostasis, metabolic function, and epithelial barrier integrity [72]. Spaceflight has been associated with changes in microbiome composition and function across multiple body sites. These alterations have been linked to immune dysregulation, metabolic changes, and impaired barrier function. At the level of the skin microbiome, spaceflight induces a marked reduction in Gammaproteobacteria and Betaproteobacteria taxa that normally contribute to pathogen exclusion and maintenance of cutaneous antimicrobial defense environments while promoting expansion of potentially opportunistic microbial populations [73]. The protective functions of these commensal organisms include competitive exclusion of pathogens, reinforcement of epithelial barrier integrity, and modulation of host antimicrobial peptide signaling [20]. Their depletion may contribute to increased susceptibility to dermatitis, delayed wound healing, and skin-associated infections during long-duration missions [73,74].

Longitudinal metagenomic analyses have identified evidence of microbial transmission between habitat-associated environmental surfaces and astronaut microbiomes, suggesting that exogenous ecological exchange within confined spacecraft environments acts as a destabilizing force distinct from and compounding physiological stress alone [75]. Consistent with this, the nasal microbiome undergoes compositional restructuring characterized by expansion of genera such as Staphylococcus, Corynebacterium, and Bifidobacterium, with shifts associated with upper airway colonization and respiratory inflammatory processes [76].

**Figure 4:**
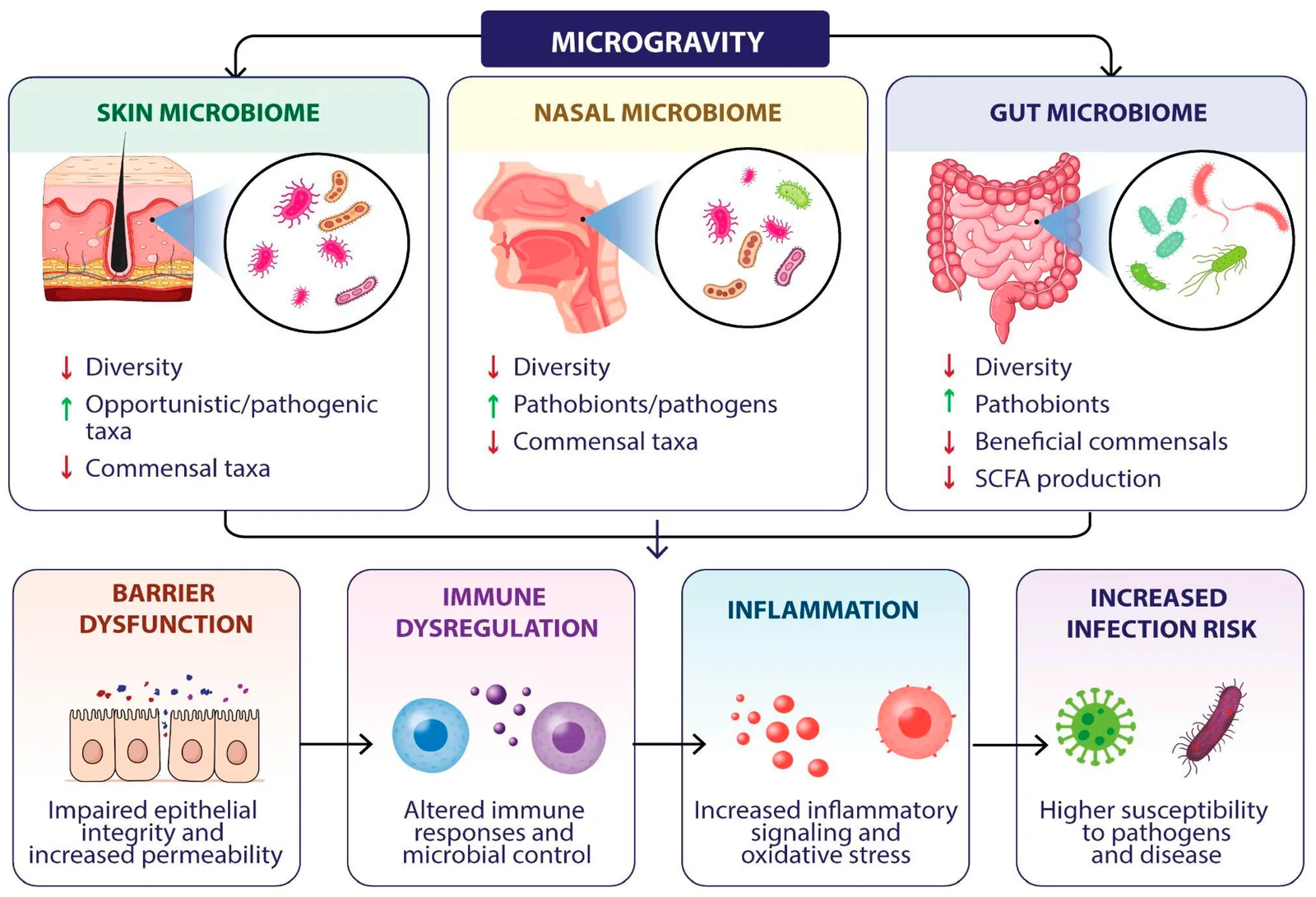
Microbiome Disruption in Spaceflight: From Sites to Health Consequences. Schematic illustration of the host–microbiome–immune axis under spaceflight conditions. Exposure to microgravity alters microbial community structure across key body sites, including the skin, nasal cavity, and gastrointestinal tract, characterised by reduced microbial diversity, depletion of beneficial commensal taxa, and expansion of opportunistic/pathobiont populations [73,75,76,77,78,79]. In the gut, these alterations are associated with changes in microbial metabolism and reduced production of beneficial metabolites, including short-chain fatty acids [77,78,79]. Collectively, site-specific dysbiosis contributes to epithelial barrier dysfunction, immune dysregulation, and chronic inflammatory signalling, potentially increasing susceptibility to infection and affecting host resilience during spaceflight [66,76,77]. Created in BioRender. Dey, S. (2026) https://BioRender.com/yj0s6a2 (accessed on 27 June 2026).

The gastrointestinal microbiome appears particularly susceptible to microgravity-associated perturbation. Several studies have reported increases in the Firmicutes/Bacteroidetes ratio together with alterations in anaerobic taxa such as Clostridiales. However, the direction and magnitude of microbiome changes are not always consistent across studies [3,77,78]. These compositional alterations are functionally significant because they may impair short-chain fatty acid production, increase intestinal permeability, and activate systemic pro-inflammatory signaling pathways [78]. Altered microbial metabolite availability, including disrupted short-chain fatty acid biosynthesis and altered tryptophan catabolism, may further modulate mucosal immune tone, epithelial barrier function, and cytokine signaling through established neuroimmune–microbial communication pathways [78]. Longitudinal multi-omic profiling from the Inspiration4 civilian spaceflight mission showed that microbial compositional changes were accompanied by immune transcriptional alterations and metabolite shifts during orbital spaceflight [79]. These findings support an interaction between the microbiome and systemic immune responses during spaceflight. The major microbiome alterations across different body sites and their potential consequences for host immunity and health are summarized in Figure 4.

The principal microbiome alterations reported during spaceflight, their proposed mechanistic drivers, and associated biomedical consequences are summarized in Table 4.

Together, these findings suggest that microgravity-induced dysbiosis and immune dysfunction may reinforce one another during long-duration missions [66,78,79]. Probiotic supplementation is considered one of the most operationally feasible near-term microbiome countermeasures, with evidence suggesting potential benefits in maintaining microbial diversity, gastrointestinal barrier integrity, and immune resilience under spaceflight-relevant conditions [80]. However, the optimal microbial composition, dosing strategy, and mission-phase timing remain incompletely defined, and clinical demonstration of these benefits during genuine long-duration orbital missions requires systematic prospective evaluation. In Saudi Arabia, this emerging field is reflected in initiatives such as BioGravity, launched by the Saudi Space Agency to cultivate national collaboration and expand research capability in biomedical microgravity science [60].

### 4.3. Agricultural and Environmental Applications

Although many enabling technologies originate from terrestrial biotechnology, microgravity-enabled biological research provides unique experimental systems for studying stress adaptation, closed-loop cultivation, and bioprocess engineering under extreme environmental conditions, thereby accelerating translational applications in agriculture and environmental sustainability [57,81,82,83]. Within the Saudi context, such advances resonate strongly with the innovation and sustainability priorities articulated under Saudi Vision 2030, particularly in food security, water sustainability, environmental resilience, and climate-adaptive agricultural systems.

In precision crop improvement, CRISPR/Cas9-mediated genome editing enables targeted modification of stress-response regulatory networks, generating cultivars with enhanced tolerance to drought, salinity, heat, and biotic stressors without introducing foreign genetic material [84]. RNA interference provides a complementary strategy through species-specific silencing of essential insect genes, enabling highly selective crop protection while minimizing ecological disruption to beneficial non-target organisms [85]. Of particular relevance to arid and saline environments, codA-transgenic tomato (Solanum lycopersicum) engineered to accumulate glycinebetaine via the choline oxidase gene from Arthrobacter globiformis demonstrates improved K^+^/Na^+^ homeostasis, reduced oxidative damage, and preserved photosynthetic efficiency under saline conditions—a model with potential relevance to Saudi coastal and arid-zone cultivation systems [86]. Saline-adapted hydroponic cultivation systems pioneered by Red Sea Farms employ seawater-based cooling and low-freshwater production strategies under extreme desert conditions, representing a practical convergence of biotechnology, water sustainability, and controlled-environment agriculture. Parallel microgravity-informed plant studies have additionally advanced closed-loop agricultural life-support engineering, with Mars analogue experiments demonstrating that biologically treated basaltic regolith and biodesalinated high-salinity water can sustain crop propagation in resource-constrained environments [81].

Environmental applications have also been explored. Genetically engineered microorganisms with enhanced metabolic pathways for degradation of petroleum hydrocarbons, heavy metals, synthetic dyes, and persistent industrial pollutants offer scalable and environmentally preferable alternatives to physicochemical remediation [87], directly aligning with Saudi industrial sustainability priorities. Environmental DNA metabarcoding has emerged as a high-sensitivity, non-invasive surveillance tool for biodiversity monitoring and ecosystem health assessment across terrestrial, coastal, and marine environments [88]. In renewable energy biotechnology, targeted modification of lignin biosynthetic pathways in sugarcane and other biomass crops enhances fermentable carbohydrate accessibility, supporting next-generation bioethanol production [89]. Meanwhile, life-support technologies originally developed for orbital missions, including advanced membrane-based water purification systems and microgravity-derived carbon dioxide separation platforms, offer promising terrestrial applications in industrial water recycling, arid-environment water recovery, and sustainable air revitalization systems [82,83]. Together, these advances support applications in sustainable agriculture, environmental resilience, and food security.

### 4.4. Translational Therapeutic and Countermeasure Opportunities

Findings from spaceflight biology have contributed to the development of countermeasures relevant to both spaceflight and terrestrial clinical settings. Microgravity-informed pathogen studies have revealed virulence signaling pathways and immunogenic targets not readily detectable under conventional terrestrial culture conditions, informing novel vaccine discovery strategies and antimicrobial countermeasure development [62,65]. Likewise, microbiome-targeted interventions, including probiotic supplementation and dietary modulation, represent promising operational strategies for preserving epithelial barrier integrity, microbial homeostasis, and immune resilience during prolonged missions [80].

Beyond direct intervention, astronaut health monitoring frameworks, including cytokine multiplex profiling, latent herpesvirus reactivation surveillance, and microbiome compositional indexing, provide transferable models for systems-level immune monitoring in immunocompromised terrestrial populations, including transplant recipients, oncology patients receiving myelosuppressive therapies, and individuals exposed to prolonged confinement or isolation [57,66]. More broadly, personalized medicine frameworks integrating genomic, epigenomic, immune, and microbiome profiling align closely with the multi-omic infrastructures emerging from international space bioscience initiatives such as NASA GeneLab and the Space Omics and Medical Atlas, suggesting a valuable translational bridge between space systems biology and terrestrial precision medicine [5,12].

At the national level, the Saudi Human Genome Program and the Genomic Medicine Center of Excellence at King Faisal Specialist Hospital and Research Centre have expanded national capabilities in genomics, biobanking, and multi-omics research [39,59]. Such infrastructure could support future investigations in space biology by enabling the generation, analysis, and interpretation of complex biological datasets relevant to human adaptation, precision medicine, and translational biomedical research.

## 5. Ethical and Legal Issues in Genetic Research

### 5.1. Overview of Ethical and Legal Issues

Saudi Arabia is advancing its genetic research with national biobanking, genomic medicine, and increasingly with cross-disciplinary fields such as space biology and microgravity research. These scientific gains are accompanied by important ethical and legal issues that remain at the centre, including informed consent, confidentiality, data governance, sample ownership, discrimination, and appropriate limits of genome modification. These issues are especially important in research involving hereditary information, children, stored biospecimens or international collaboration, as the scientific value of the data is matched by an increased obligation to protect participants and their families [90,91].

#### 5.1.1. Informed Consent

In Saudi research ethics, informed consent is a fundamental requirement, not a procedural formality. Consent for genetic and biobank studies should include information about the purpose of the research, the possibility of future reuse of samples and data, the duration of storage, and the participant’s right to withdraw. This is particularly relevant because genetic research often generates information that may be relevant long after the original research has been completed, and because participants may not be able to foresee all future uses of their samples at the time of donation. Saudi Biobank governance in research with children emphasizes parental permission, child assent where appropriate, and special consideration of confidentiality and guardianship [92,93,94].

#### 5.1.2. Privacy, Confidentiality, and Discrimination

Genetic data are sensitive because they may reveal information about disease susceptibility, ancestry, reproductive risks, and relatives. Saudi biobank governance and the Law of Ethics of Research on Living Things emphasize protection of participants, confidentiality, and secure management of biological and genetic information.

Researchers should apply robust de-identification or coding procedures, secure storage, role-based access controls, controlled data retrieval, and documented rules for data export. These safeguards are especially important in Saudi and Gulf settings, where genetic information may have social implications for family relationships, marriage, employment, insurance, and social reputation. Ethical oversight should therefore ensure that genetic information is used only within the approved research scope and does not expose participants or families to direct or indirect discrimination [92,93,94,95].

#### 5.1.3. Sample Ownership, Secondary Use, and Sharing

Stored genetic material may support important future research, but secondary use raises questions about participant expectations, governance authority, and whether the proposed use remains within the original consent. Saudi regulations require ethical oversight of biological materials, including their documentation, retrieval, storage, and export.

Research protocols should define who controls access to samples, who authorizes secondary analyses, whether additional ethical review or consent is needed, and how data and samples may be shared internationally. Material-transfer and data-sharing agreements should specify permitted uses, security standards, publication arrangements, restrictions on onward transfer, and procedures for disposal or return where applicable [91,93,94].

#### 5.1.4. Islamic Bioethics

Islamic bioethics provides an important interpretive framework for genetic research in Saudi Arabia. Its assessment commonly considers public benefit, prevention of harm, human dignity, justice, and preservation of lineage. These principles can guide decisions concerning consent, confidentiality, guardianship, family interests, and the acceptable purposes of genetic interventions [96,97].

### 5.2. Saudi Regulation and Microgravity-Specific Gaps

The legal framework in Saudi Arabia provides a structured basis for managing these concerns, but there are significant gaps. The National Committee of Bioethics has published standards and regulations such as models for informed consent in genetic research and guidelines for the export of biological samples. The Law of Ethics in Research on Living Things also sets up mechanisms for ethical supervision and a national database for genetic material. However, studies of Saudi and regional genomic ethics have noted the uneven practical implementation of these rules and suggested that more detailed guidance is needed on issues such as genetic discrimination, international data sharing, and molecular epidemiology. These gaps are particularly visible in fast-moving fields like microgravity genetics. The empirical basis for the consent problem is now well-established: longitudinal assessments of astronauts on International Space Station missions of a year’s duration have documented telomere elongation, increased numbers of short telomeres post-flight, DNA methylation changes in immune and oxidative stress-related pathways, and persistent disruption of transcription in a subset of genes even six months after return to Earth [3]. Against this biological backdrop, three particular lacunae in governance are worthy of special attention. First, jurisdictional ambiguity: biological samples collected from Saudi astronauts on the International Space Station are governed by the domestic law of the collecting agency under the ISS Intergovernmental Agreement, not Saudi law, but Saudi biobank export rules do not specifically cover this scenario [98,99]. Second, secondary-use consent. Existing consent templates were designed for ground-based biobank studies and do not consider the possibility that orbit-derived samples may subsequently reveal clinical findings relevant to the participant’s family members. Third, data sharing compatibility: NASA and ESA open science data policies assume broad public release [100], but Saudi regulations restrict the export of genetic material, which could conflict with these requirements. Filling these three gaps would mean dedicated additional guidance from the National Committee of Bioethics on space-specific contexts, experiments involving international partners, astronaut-derived samples, and complex cross-border data flows [33,47,94,101].

#### Microgravity-Specific Consent Challenges

General genetic-research consent should be supplemented, rather than duplicated, when samples are collected during spaceflight or used in microgravity studies. This additional consent should address the uncertain long-term significance of findings obtained under spaceflight conditions and the potential scientific value of astronaut-derived samples, which may create pressure for extensive future reuse. Participants should be informed whether their samples and data may be stored in national or international biobanks, reused in later space-health studies, or shared with foreign institutions. Consent should also address the possibility of findings with personal or familial relevance, the proposed approach for communicating such findings, the applicable jurisdiction, conditions of data access, and limits on international transfer. These measures are important because space research often involves small participant numbers, multinational partners, and complex custody arrangements for samples and data [47,102,103].

### 5.3. Balancing Innovation and Protection

The governance of genetic and microgravity research in Saudi Arabia should balance scientific innovation with protection of dignity, privacy, justice, lineage, and participant welfare. For microgravity biomedical research, this requires context-sensitive consent, secure data and sample governance, transparent rules for international collaboration, and ethical review that remains responsive to the distinctive conditions of spaceflight. Such a framework would support the development of Saudi space genetics while ensuring that technological progress does not outpace the legal and ethical infrastructure required for responsible research [3].

### 5.4. Germline Editing and Emerging Technologies

Detailed consideration of germline editing and emerging editing technologies is beyond the immediate scope of current microgravity genetic research. Nevertheless, interventions that could produce heritable genetic changes raise distinct ethical concerns, including uncertain long-term safety, effects on future generations who cannot consent, possible implications for lineage, and risks of non-therapeutic enhancement. Any future application of heritable editing in a space-related context would require exceptional scientific justification, explicit legal authorization, and rigorous national and international ethical oversight [103,104].

The principal genome-editing technologies, together with their ethical considerations, Islamic bioethical perspectives, and potential relevance to Saudi microgravity research, are summarized in Table 5.

### 5.5. Global and Islamic Perspectives on Heritable Editing

International governance frameworks emphasize the need for transparent institutional, national, and global oversight of human genome editing, particularly when interventions are heritable. From an Islamic bioethical perspective, therapeutic interventions may be viewed more favorably than interventions intended for enhancement; however, heritable editing remains ethically challenging because of concerns regarding safety, necessity, dignity, and lineage. Mitochondrial replacement therapy similarly raises questions regarding parentage and lineage, as well as unresolved safety concerns, and would require careful ethical and legal assessment before any future application [105,106,107,108].

### 5.6. Somatic Editing for Astronauts

Somatic genome editing does not affect future generations but may raise important ethical issues if considered for astronaut health. Its ethical acceptability would depend on whether it is intended to prevent or treat a serious health risk, whether adequate evidence of safety and benefit exists, and whether less invasive alternatives are available. In contrast, interventions intended primarily to improve performance beyond the level required for health protection or mission safety would be more difficult to justify. Saudi space-medicine governance should therefore assess any proposed somatic editing case by case, with particular attention to safety, long-term uncertainty, voluntariness in an employment-related setting, fairness, and the distinction between therapy and enhancement [97,109].

## 6. Biological Mechanisms and Methodological Challenges in Microgravity Research

### 6.1. Technological Challenges in Microgravity Experiments

Despite the experimental opportunities provided by orbital research platforms, spaceflight biology remains limited by engineering constraints, complex experimental environments, and incomplete analytical standardization, which continue to affect reproducibility and data interpretation [1,110]. Although technological innovation has substantially expanded experimental access to space-based biology, several methodological barriers continue to limit the fidelity, scalability, and interpretability of microgravity research.

One of the foremost challenges lies in the intrinsic operational constraints of orbital experimentation. Biological payloads used in spaceflight experiments are restricted by limitations in mass, volume, power consumption, and crew interaction time, requiring miniaturized systems and simplified experimental designs [110]. While these platforms have enabled increasingly sophisticated biological studies, restricted throughput, limited onboard flexibility, and small cohort sizes continue to constrain replicate design and reduce statistical robustness—particularly in multi-omic investigations, where biological heterogeneity requires larger sample numbers for meaningful inference [110,111].

Compounding these limitations, biological perturbation may begin before orbital microgravity exposure itself. Launch-associated dynamic vibration and static hypergravity have been shown to independently induce measurable molecular alterations, indicating that experimental perturbation is initiated at lift-off rather than only after orbital insertion [112]. Subsequent transit, storage, and delayed sample processing introduce additional layers of experimental noise, including RNA degradation, metabolomic instability, and disruption of microbial community structure. These pre-analytical variables are not always adequately accounted for in downstream analyses, which may introduce systematic bias and complicate interpretation of the biological findings [112].

Another major challenge is that spaceflight biology involves multiple simultaneous environmental stressors. Microgravity does not operate as an isolated variable in true orbital conditions; rather, biological systems are simultaneously exposed to ionizing radiation, altered fluid convection, circadian disruption, oxidative stress, and mission-specific operational stressors, making unambiguous causal inference exceptionally difficult [1,3]. Experimental studies suggest that combined spaceflight stressors may produce biological effects that differ from those caused by individual exposures alone. Barravecchia et al. (2021) [113] demonstrated that microgravity and space radiation may exert either opposing or synergistic effects on autophagy-associated molecular pathways depending on cellular context. Likewise, Beheshti et al. (2021) [114] using simulated microgravity ground models combined with radiation exposure, reported that combined stressors promote genomic instability, epigenetic remodeling, and mitochondrial dysfunction through mechanisms distinct from either insult alone—findings that warrant prospective validation under genuine orbital conditions. Extending this concept, Wadhwa et al. (2024) [7] further showed that radiation–microgravity interactions produce highly context-dependent effects on cytokine signaling, lymphocyte activity, and innate immune regulation, underscoring substantial uncertainty in mechanistic risk modeling for long-duration missions. Although ground-based analogues, including clinostats, rotating wall vessels, and random positioning machines, remain experimentally tractable, simulated microgravity should be clearly distinguished from genuine orbital microgravity. These platforms reproduce selected aspects of gravitational unloading but do not replicate the complete spaceflight environment, which includes ionizing radiation, launch-associated forces, altered fluid dynamics, circadian disruption, and mission-specific operational stressors. Findings obtained under simulated microgravity should therefore be interpreted as model-based approximations rather than direct equivalents of orbital spaceflight and, where possible, validated under genuine microgravity conditions [110,111].

Recent advances in onboard molecular analysis are beginning to address some of these limitations. In-flight nanopore DNA sequencing, RNA extraction, quantitative PCR, and culture-independent microbial profiling have demonstrated the feasibility of increasingly autonomous molecular analysis during orbital missions [115,116,117], marking a shift from passive sample banking toward more dynamic, hypothesis-driven orbital bioscience.

Standardized analytical frameworks and shared biological data infrastructure have also expanded in recent years. Rutter et al. (2020) [118] established the International Standards for Space Omics Processing framework, proposing harmonized protocols for sample handling, metadata annotation, and bioinformatic standardization across missions. Building upon this foundation, the Space Omics and Medical Atlas (SOMA) published in Nature in 2024 integrated multi-omic, clinical, and cellular datasets across multiple crewed missions, representing a more than tenfold increase in publicly available human spaceflight omics data and establishing a new precedent for coordinated multi-mission astronaut biobanking [5]. Complementing this effort, NASA GeneLab has consolidated transcriptomic, proteomic, metabolomic, and epigenomic datasets from hundreds of spaceflight experiments into a curated open-access repository, enabling systems-level reanalysis and cross-species pathway comparison independent of individual mission datasets [12].

Nevertheless, important methodological gaps remain. Closing these barriers will require automated closed-loop bioreactors with integrated real-time molecular readouts, AI-assisted adaptive payload management, and orbital validation of single-cell and spatially resolved transcriptomic platforms whose biological resolution substantially exceeds current bulk-tissue approaches [5,119]. These advances will be important for improving the reproducibility and biological interpretation of microgravity research and may strengthen its applications in astronaut health and terrestrial biomedical research.

### 6.2. Mechanistic Insights into Microgravity-Induced Biological Changes

Understanding the biological effects of microgravity requires investigation of the molecular pathways involved in cellular adaptation, dysfunction, and disease susceptibility during spaceflight. Emerging evidence indicates that the biological consequences of microgravity do not arise from isolated pathways, but instead emerge from coupled disturbances in cellular mechanotransduction, redox homeostasis, mitochondrial function, epigenetic regulation, immune signaling, and host–microbiome dynamics forming an integrated systems-biology network that underpins physiological adaptation to spaceflight [1].

One of the earliest and most pervasive responses to microgravity is disruption of cellular mechanotransduction, which alters cytoskeletal organization, membrane tension, focal adhesion signaling, and intracellular force transmission. These biomechanical disturbances propagate downstream molecular effects that include altered calcium signaling, mitochondrial electron transport dysfunction, and increased reactive oxygen species (ROS) generation [1]. Elevated reactive oxygen species (ROS) contribute to DNA strand breaks, oxidative base damage, and protein oxidation, while activating stress-response pathways involving NF-κB, p53, and MAPK signaling. These pathways are known to regulate inflammation, cell-cycle control, and apoptosis under conditions of cellular stress [1,120]. Persistent oxidative stress may also impair DNA repair processes through oxidative modification of repair proteins and disruption of damage-response signaling pathways. Together, these effects may contribute to the accumulation of genomic damage during prolonged spaceflight, particularly when radiation exposure and microgravity occur simultaneously [113,114].

Mitochondrial dysfunction is closely linked to oxidative stress and is thought to contribute to several biological effects observed during spaceflight. Mitochondria are a major source of intracellular ROS and are themselves vulnerable to oxidative damage. Impairment of mitochondrial function can disrupt oxidative phosphorylation, reduce ATP production, and alter cellular signaling pathways involved in apoptosis and innate immunity [1]. Consistent with this mechanism, the NASA Twins Study identified coordinated alterations in mitochondrial metabolism, inflammatory signalling, and systemic metabolic regulation during long-duration spaceflight [3].

In addition to oxidative and metabolic changes, spaceflight appears to influence epigenetic regulation. Altered gravity conditions have been associated with changes in DNA methylation, histone modification, chromatin organization, and non-coding RNA expression [1,114]. These observations suggest that biological adaptation to spaceflight may be mediated not only by genetic damage but also by reversible changes in gene regulation.

These epigenomic shifts influence regulatory programs governing oxidative defense, tissue remodeling, immune competence, and cellular stress adaptation. Importantly, longitudinal astronaut datasets indicate that some transcriptional and epigenomic perturbations show only partial normalization following return to Earth, raising the mechanistically significant possibility that prior spaceflight exposure may encode persistent biological memory with implications for cumulative long-term health risk in career astronauts [3]. This persistence underscores the inadequacy of single post-flight assessments for capturing the full biological legacy of orbital exposure.

At the systems level, immune dysregulation and host–microbiome disruption appear to represent interconnected downstream consequences of these molecular perturbations. As discussed in Section 4.1 and Section 4.2, spaceflight-associated changes in immune function and microbial ecology frequently occur concurrently. Mechanistically, these observations suggest bidirectional interactions among redox imbalance, mitochondrial dysfunction, inflammatory signalling, epithelial barrier function, and microbial metabolism, although the directionality and causal hierarchy of these relationships remain unresolved [66,67,68,73,78,79].

Together, these findings suggest that oxidative stress, mitochondrial dysfunction, epigenetic alteration, immune dysregulation, and microbiome changes are closely interconnected rather than fully independent biological processes. An important remaining challenge is to establish causal relationships among these interconnected biological processes, as the directionality, pathway hierarchy, and reversibility of these interactions remain poorly understood in human spaceflight biology. Addressing this challenge will require mechanistically informed perturbation studies, longitudinal multi-omic sampling with controlled temporal resolution, and systems-level causal modeling integrated within emerging space-omics infrastructures such as the Space Omics and Medical Atlas (SOMA) and NASA GeneLab [5,12].

## 7. Advanced Technologies and Collaborative Opportunities in Microgravity Research

Saudi Arabia has recently increased its engagement in microgravity and space bioscience research through a series of initiatives led by the Saudi Space Agency (SSA) and collaborations with national and international partners. These activities have focused on building research capacity, supporting space-related biomedical studies, and establishing the infrastructure needed for future participation in space life sciences. This section highlights current initiatives and discusses areas where further collaboration may accelerate the development of microgravity research within the Kingdom.

A notable step was the launch of the BioGravity initiative by the Saudi Space Agency in October 2024. The initiative was established to support biomedical and biotechnology research conducted under microgravity conditions while creating opportunities for collaboration among researchers from universities, research centres, and industry [31,60]. Led by Saudi astronaut Rayyanah Barnawi, BioGravity functions as a national community of practice that facilitates knowledge exchange, training activities, and project development in space biosciences. Since its establishment, the initiative has organized workshops and networking activities aimed at connecting Saudi researchers with international experts and identifying research priorities relevant to human health and biotechnology in space [31,60]. In parallel, the SSA has introduced mechanisms that allow researchers to submit proposals for microgravity-related investigations through national research platforms and open calls [121].

Saudi Arabia’s participation in the Axiom-2 mission to the International Space Station in 2023 marked the country’s first involvement in human spaceflight research. During the mission, astronauts Rayyanah Barnawi and Ali AlQarni conducted a series of biomedical experiments examining physiological and molecular responses to spaceflight [122]. According to information presented by the SSA to the United Nations Office for Outer Space Affairs, these investigations included measurements of telomere dynamics, neurological biomarkers, brain electrical activity, cerebral perfusion, optic nerve sheath diameter, and intracranial pressure-related parameters [122]. Although limited in duration, these experiments generated initial data from Saudi-led studies in space and demonstrated the feasibility of conducting biomedical research during crewed missions.

Collaboration with healthcare institutions has also become part of the national strategy for developing space medicine research. In 2025, the Saudi Space Agency and King Faisal Specialist Hospital and Research Centre signed a memorandum of understanding covering astronaut healthcare, biotechnology research under microgravity conditions, education, and scientific collaboration [123]. The agreement established a framework for joint projects and working groups while supporting medical monitoring before, during, and after space missions. Given KFSHRC’s expertise in stem cell biology, genomics, oncology, neurosciences, and cardiovascular medicine, the partnership may support future translational applications arising from spaceflight-related biomedical research.

The SSA has additionally announced plans for dedicated infrastructure and workforce-development programmes to support future space bioscience activities [122]. These include the development of a national space innovation laboratory and initiatives aimed at strengthening expertise in space-related biomedical research. Educational programmes such as the SSA Microgravity Challenge, conducted in collaboration with Axiom Space, have introduced students to microgravity experimentation through projects linked to the International Space Station and related engineering activities [124]. Such programmes may contribute to developing a future workforce with experience in space biotechnology, biomedical engineering, and translational health research.

International experience offers valuable templates for the design of Saudi research collaborations. NASA’s Human Research Program has identified pharmaceutical stability, efficacy, and on-demand manufacturing during long-duration spaceflight as priority research areas, and considers medications the primary countermeasure for many spaceflight-related health risks [125,126]. Recent collaborations between NASA and academic pharmacy research groups, such as the UCL School of Pharmacy and FABRX, illustrate how interdisciplinary partnerships between space agencies and university-based pharmaceutical scientists can accelerate translational innovation in 3D-printed personalized medicines and on-demand drug manufacturing for space missions [127]. Comprehensive reviews of pharmaceutical supply for exploration spaceflight further highlight the challenges of drug stability, storage, formulation, and the need for personalized therapeutics under spaceflight conditions [126]. Such models provide direct precedents for engaging Saudi pharmacy schools, clinical pharmacology groups, and the Saudi Food and Drug Authority (SFDA) in space pharmaceutical research and regulatory science.

In parallel with academic collaborations, international initiatives led by NASA and the ISS National Laboratory have demonstrated the growing translational and economic value of microgravity-enabled pharmaceutical research, tissue engineering, and in-space biomanufacturing [128,129,130]. The In-Space Production Applications (InSPA) initiative supports scalable, commercially viable manufacturing in low Earth orbit, including pharmaceutical production, advanced biomaterials, and regenerative medicine [129,130]. Recent NASA-supported InSPA awards have funded projects on biomanufacturing of drug-delivery devices, expansion of hematopoietic stem cells, stem cell therapies, volumetric additive manufacturing for organ production, and pharmaceutical in-space laboratories [131]. Microgravity environments may offer distinctive advantages for these applications, including reduced sedimentation, altered crystal growth, improved structural organization, and enhanced tissue assembly, with established benefits for protein crystallization, nanomedicine, and bioprinted tissue constructs [132,133].

These international models provide strategic direction for Saudi Arabia in localizing advanced pharmaceutical and biotechnology industries aligned with Vision 2030 objectives related to innovation, advanced manufacturing, and economic diversification. The Kingdom’s growing investments in biotechnology, additive manufacturing, artificial intelligence, and precision medicine create favourable conditions for establishing specialized research centres, translational innovation hubs, and biotechnology startups focused on space-derived healthcare technologies. Existing national infrastructure already provides a foundation for such an ecosystem, including the Interdisciplinary Research Center for Aviation and Space Exploration (IRC-ASE) at King Fahd University of Petroleum and Minerals (KFUPM), which operates as a formal SSA partner and coordinates national activities in space systems, nano-satellites, and applied space research [133]. National research universities such as King Saud University, King Abdulaziz University, and King Abdullah University of Science and Technology (KAUST), together with research bodies such as the King Abdulaziz City for Science and Technology (KACST), represent further natural partners for translational space bioscience programmes. Engagement of the Saudi Food and Drug Authority (SFDA) in regulatory science for in-space pharmaceutical production would further support the development of a coherent national ecosystem capable of supporting both terrestrial healthcare applications and future long-duration missions.

These initiatives indicate that Saudi Arabia is developing an integrated ecosystem for microgravity and space biomedical research through coordinated activity among governmental agencies, healthcare institutions, universities, and international space partners. The growing emphasis on biotechnology, precision medicine, artificial intelligence, and translational innovation under Vision 2030 creates a credible pathway for the Kingdom to emerge as a regional contributor to space medicine and microgravity biosciences, particularly if national strengths in clinical research, pharmaceutical sciences, and advanced manufacturing are coordinated through long-term, well-defined collaborative frameworks [133].

### 7.1. Advanced Models in Microgravity Research

Advanced experimental models have become essential tools in microgravity and space bioscience research because conventional two-dimensional cell cultures and animal models often fail to reproduce the complex physiological responses associated with spaceflight. Recent advances in organoid systems, organ-on-a-chip technologies, bioprinting approaches, and ground-based microgravity simulators have enabled researchers to better mimic tissue architecture, mechanobiological signaling, and cellular interactions under microgravity conditions [110,134,135,136]. These platforms provide improved physiological relevance, enhanced control over cellular microenvironments, greater experimental reproducibility, and increased translational potential for applications in regenerative medicine, astronaut health, disease modeling, and precision therapeutics compared with traditional experimental systems [110,134,135]. The following sections review the principal advanced model systems currently employed in space bioscience, such as organoids and spheroids, organ-on-a-chip and microphysiological systems, hybrid bioprinted platforms, ground-based simulators, and emerging orbital platforms, highlighting their applications, comparative advantages, and remaining limitations.

Importantly, findings across these platforms should not be treated as interchangeable, since real microgravity (orbital spaceflight and sample return) and simulated microgravity (parabolic flights, clinostats, random positioning machines, and rotating wall vessels) generate distinct mechanical and environmental conditions, each with characteristic experimental limitations.

Three-dimensional organoids and multicellular spheroid cultures are widely used biological models in space bioscience. Organoids are stem-cell-derived self-organizing structures that recapitulate key architectural and functional features of native tissues, including the brain, intestine, liver, and heart [137,138]. Unlike two-dimensional monolayer cultures, organoids preserve cell–cell communication, extracellular matrix interactions, and tissue-specific microenvironments, enabling more accurate modeling of cellular adaptation to microgravity [136,137,139]. Recent studies have shown that microgravity aboard the ISS can alter bone remodeling and stem cell behavior, making organoid and spheroid systems valuable platforms for investigating musculoskeletal deterioration and tissue regeneration during spaceflight [140,141].

Beyond single-tissue organoids, assembloids generated through the fusion of multiple organoid types allow investigation of inter-organ communication and systemic adaptation, although their application under true microgravity remains an emerging area [142]. Multicellular tumor spheroids exposed to simulated microgravity have likewise shown altered aggregation, migration, invasion, and mechanotransduction, indicating that gravitational forces influence cancer progression and cellular biomechanics [143]. Despite these strengths, organoid-based systems still face important limitations, including incomplete cellular heterogeneity, variability among stem cell lines, restricted long-term maturation, absence of vascularization, and technical challenges associated with maintaining viable cultures during and after orbital experiments [139,142].

Organ-on-a-chip and microphysiological systems (MPS) represent another rapidly advancing platform in space biomedical research. These microfluidic devices reproduce tissue-level environments through controlled fluid flow, biochemical gradients, multicellular architecture, and mechanical stimulation. In contrast to static cultures, they allow continuous monitoring of cellular responses while supporting integration of multiple tissue compartments into interconnected microphysiological platforms [144,145,146,147]. Their compact size, automation potential, and reduced reagent requirements make them particularly suitable for spaceflight experiments, where hardware volume, payload mass, and crew intervention are limited [146,147]. The broader field has also evolved toward linked multi-organ systems, real-time biosensors, vascularized tissue models, and platforms designed for drug discovery, toxicity testing, and personalized medicine. NIH and CASIS tissue-chip collaborations with the ISS National Laboratory have shown that MPS can be adapted for launch, spaceflight, ISS integration, and return, while improving robustness, miniaturization, throughput, and autonomous operation suitable for crewed missions [146].

Microgravity-on-a-Chip (MOC) platforms represent a more specialized subclass of microfluidic systems developed specifically to overcome technical limitations of conventional ground-based simulators [111,143]. Random positioning machine (RPM) experiments typically rely on fully filled T25 flasks or sealed well plates to minimize air bubble formation during rotation, but these approaches require large volumes of culture medium and can introduce shear stress artefacts, inconsistent oxygen diffusion, and residual centrifugal effects that confound biological interpretation. MOC systems address these issues by using sealed PDMS-based microfluidic chambers requiring only microliter-scale media volumes while preventing air bubble formation, leakage, and excessive shear stress during rotation. The gas-permeable properties of PDMS additionally improve oxygen exchange and nutrient diffusion, supporting more stable cellular microenvironments during long-duration simulated microgravity exposure [111].

Bioprinting and biofabrication technologies have emerged as important tools for space medicine and regenerative research. Microgravity provides unique conditions for tissue assembly because reduced sedimentation and gravitational collapse facilitate the fabrication of complex three-dimensional biological structures [134,136,148]. Bioprinting enables the generation of tissue analogues, organoid-like structures, and scaffold-free constructs with improved spatial organization and cell viability [148,149], and the International Space Station currently hosts biofabrication capabilities for manufacturing tissue constructs under true microgravity [134,136]. Disruptive approaches such as magnetic levitation biofabrication, scaffold-free volumetric bioprinting, and acoustic-assisted cellular assembly are also being explored to improve tissue complexity and more accurately mimic native physiological architectures [150]. Current limitations include restricted bioink formulations, limited print resolution at clinically relevant scales, and difficulties in achieving functional vascularization and long-term maturation of printed constructs [148,151].

Building on these advances, hybrid systems that combine bioprinting with organ-on-a-chip platforms have further increased the complexity of microgravity disease modeling [143,146,148]. Conventional organ-on-a-chip systems reproduce biomechanical forces such as vascular shear stress but often depend on simplified, artificially seeded cellular arrangements, whereas bioprinted constructs offer superior spatial organization and extracellular matrix mimicry yet typically lack dynamic perfusion [148,151]. Hybrid bioprinted MPS overcome these limitations by integrating vascularized tissue fabrication with microfluidic perfusion networks capable of reproducing active blood flow, nutrient transport, and tumor–vascular interactions under simulated microgravity. For example, vascularized glioblastoma-on-a-chip systems allow continuous monitoring of tumor self-organization, mechanotransduction, and microenvironment remodeling from the earliest stages of tumor formation, rather than relying on pre-formed spheroids [143].

Ground-based microgravity simulators remain indispensable because access to orbital facilities is limited, highly competitive, and financially demanding. Clinostats, RPMs, rotating wall vessels (RWVs), low-shear modeled microgravity bioreactors, parabolic flight systems, drop towers, and newer scalable microgravity simulators provide accessible methods for investigating cellular responses, microbial adaptation, mechanotransduction pathways, and tissue remodeling under simulated or transient microgravity conditions [110,152,153]. RWVs generate low-shear, low-turbulence environments that support long-term culture and three-dimensional tissue or organoid formation, although uneven cell distribution and residual shear stress may still occur [152]. RPMs and clinostats produce simulated microgravity by continuously changing the direction of the gravity vector but can introduce shear forces, vibration, residual acceleration, and sample-volume limitations [152]. More recent scalable simulators improve on these designs through optimized algorithms, equal treatment of multiple culture flasks, incubator-compatible footprints, and long-term culture capability for tissue engineering applications. Validation studies with bone marrow stem cells and skeletal muscle myoblasts have shown that such systems produce biological responses comparable to RPM or RWV platforms, supporting their use for reproducible musculoskeletal and regenerative medicine studies [153].

While ground-based simulators provide accessible and reproducible tools for hypothesis generation and screening, validation under true microgravity remains essential, motivating continued development of orbital experimental platforms. Although the ISS remains the most established environment for long-term experiments and sample return, emerging platforms such as CubeSats, SmallSats, and the Lunar Orbital Gateway offer complementary opportunities for miniaturized, automated, and longer-duration biological experiments beyond low Earth orbit [154]. These platforms are particularly relevant for studying the combined effects of microgravity, radiation, and other environmental stressors that cannot be fully reproduced on Earth. In situ analytical systems may further reduce dependence on pre- and post-flight analysis by enabling real-time molecular, cellular, and biochemical measurements during missions. Nevertheless, careful experimental design remains critical because no ground-based simulator fully reproduces the spaceflight environment, including cosmic radiation, altered fluid dynamics, launch vibration, confinement, circadian disruption, and prolonged gravitational unloading [154].

A comparative summary of the principal advanced model systems used in microgravity research is provided in (Table 6). Each platform offers distinct advantages and limitations, and the choice of model should be guided by the biological question, available infrastructure, and intended translational endpoint.

These advanced experimental platforms are transforming microgravity research from largely descriptive observational studies toward increasingly mechanistic, predictive, and translational space biomedicine. The integration of organoids, microphysiological systems, hybrid bioprinted platforms, scalable simulators, and emerging orbital infrastructure provides new opportunities to investigate human physiology, disease progression, tissue regeneration, and therapeutic responses under reduced-gravity conditions. Continued advances in automation, miniaturization, in situ analytics, and artificial intelligence–assisted image analysis, phenotyping, and experimental design are expected to further enhance the reliability, physiological relevance, and translational impact of future space bioscience research.

### 7.2. Multi-Omics and Systems Biology Approaches

Multi-omics refers to the integration of multiple biological data layers, including genomics, transcriptomics, proteomics, metabolomics, epigenomics, and microbiomics, combined to characterize how organisms respond to conditions such as microgravity. Systems biology extends these approaches through computational and network-based analyses that model interactions among genes, proteins, metabolites, and cellular pathways, enabling prediction of physiological adaptations and disease risks during spaceflight [155]. Applications of multi-omics to microgravity research span both real and simulated microgravity platforms. A notable example of spatial multi-omics application in microgravity research involved glioblastoma organoids cultured aboard the International Space Station [156]. Researchers integrated transcriptomic profiling, secretomics, and infrared laser scanning microscopy to identify spatial gradients in inflammatory, mesenchymal, lipid, and protein-associated signatures [156]. The study demonstrated that microgravity promoted more physiologically relevant tumor architecture and immunosuppressive microenvironments compared with terrestrial controls, supporting the growing role of orbital systems biology in cancer modeling [156]. Multiple studies have shown that spatial omics preserves tissue architecture, allowing investigation of cell–cell communication and tissue remodeling under altered gravity environments [34,157,158]. Additionally, single-cell sequencing allows researchers to analyze cellular heterogeneity under microgravity at unprecedented resolution [159]. These technologies can identify cell-type-specific adaptations in tissues such as muscle, bone, and the immune system. Multi-omics integration can reveal how microbial shifts influence immunity, inflammation, and metabolic regulation during space missions [65]. Together, these approaches support precision medicine and astronaut health monitoring during long-duration spaceflight.

Several limitations, however, currently constrain the interpretation of space-derived multi-omics datasets. Sample sizes remain small owing to restricted crew availability and payload capacity, and mission-to-mission heterogeneity in sample handling, storage, and analytical pipelines introduces batch effects that complicate cross-study comparison. Integration across genomic, transcriptomic, proteomic, and metabolomic layers is technically demanding and requires harmonized reference datasets, standardized annotation frameworks, and reproducible computational pipelines, which are still under development for space-relevant applications. Furthermore, findings obtained under simulated microgravity may not directly translate to true spaceflight conditions, and cautious interpretation is required when integrating datasets generated across different experimental platforms.

### 7.3. Artificial Intelligence and Computational Modeling

Artificial intelligence (AI) and machine learning (ML) are increasingly used in microgravity research to analyze high-dimensional transcriptomic and multi-omics datasets generated from spaceflight experiments. Recent studies demonstrate that ML models can successfully classify space-flown versus ground-control biological samples by integrating heterogeneous transcriptomic datasets, highlighting the value of data harmonization approaches in space biology research [160,161]. In addition, AI frameworks have been applied to NASA Open Science Data Repository datasets to identify key molecular drivers of metabolic reprogramming under microgravity, revealing biologically meaningful gene signatures associated with tissue adaptation during spaceflight [162]. AI has become essential for analyzing large-scale multi-omics datasets generated during space biology experiments. By integrating multi-omics data with deep learning models, researchers can enable multi-scale predictive modeling of genotype–environment–phenotype relationships [163]. Beyond classification tasks, AI/ML methods are also being used to uncover hidden patterns in astronaut omics data, including immune dysregulation, mitochondrial dysfunction, and stress-response alterations that are not easily detectable using traditional statistical methods [164]. Furthermore, recent NASA-focused computational frameworks emphasize the development of “precision space health” systems that integrate multi-modal biological data, predictive modeling, and autonomous analytics to support real-time decision-making during long-duration missions [165]. The NASA Twins Study is an example of AI-driven multi-omics integration used to analyze transcriptomic, epigenomic, and proteomic changes during spaceflight [3]. Collectively, these advances demonstrate that AI-driven computational modeling provides a powerful approach for interpreting complex space biology datasets and predicting human physiological adaptation to microgravity.

## 8. Conceptual Framework of Microgravity Effects

**Figure 5:**
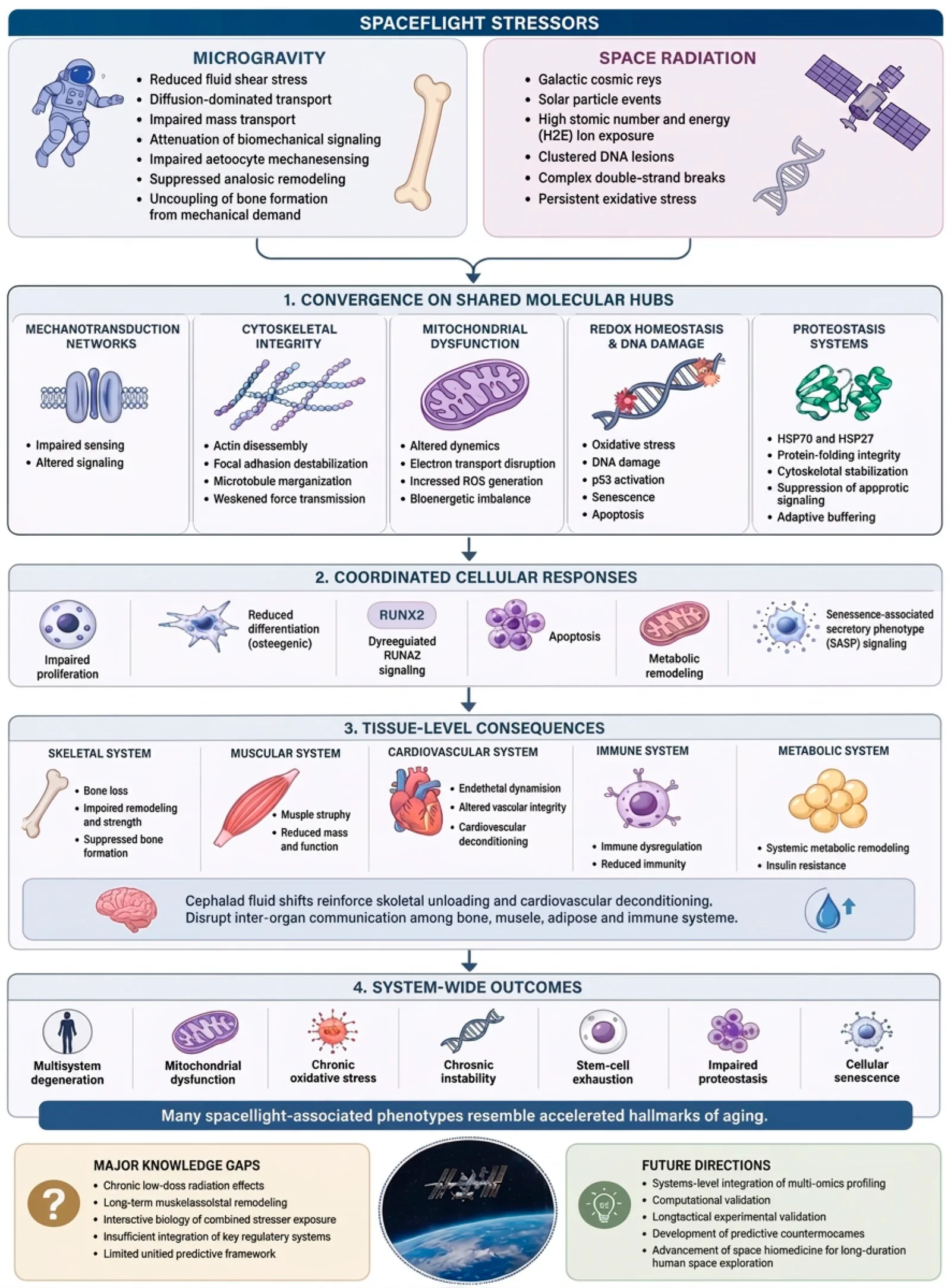
Conceptual Framework of Microgravity Effects. Microgravity and space radiation initiate interconnected molecular disturbances involving mechanotransduction, mitochondrial dysfunction, oxidative stress, DNA damage, and proteostasis. These alterations trigger coordinated cellular responses that lead to tissue dysfunction across the skeletal, muscular, cardiovascular, immune, and metabolic systems, ultimately contributing to systemic aging-related phenotypes. The framework also highlights current knowledge gaps and future research priorities for advancing space biology and precision medicine. Figure created using Microsoft PowerPoint for Microsoft 365 and FigureLabs during an active paid subscription, with authorization for academic publication.

Spaceflight exposes biological systems to an integrated stress environment in which microgravity and space radiation are physically distinct but mechanistically convergent drivers of systemic degeneration. Rather than acting independently, they converge on regulatory hubs including mechanotransduction, mitochondrial metabolism, redox homeostasis, DNA damage responses, and proteostasis, amplifying dysfunction across molecular, cellular, tissue, and organismal scales (Figure 5).

Microgravity shifts tissues from convection-driven to diffusion-dominated transport, reducing fluid shear stress, mass transport, and biomechanical signaling. In bone, reduced interstitial fluid flow through the lacunar–canalicular network impairs osteocyte mechanosensing and anabolic remodeling, uncoupling bone formation from mechanical demand [166,167,168,169,170,171,172]. Conversely, galactic cosmic rays, solar particle events, and high atomic number and energy (HZE) ions induce clustered DNA lesions, complex double-strand breaks, and persistent oxidative stress during chronic exposure [173,174].

The cytoskeleton is a principal mechanobiological interface linking physical stress to cellular adaptation. Mechanical unloading promotes actin stress-fiber disassembly, focal adhesion destabilization, and microtubule reorganization, weakening integrin-mediated force transmission and disrupting focal adhesion kinase (FAK), RhoA/ROCK, MAPK, and Hippo–YAP/TAZ signaling [175,176]. Altered mechanosensitive ion-channel activation, including PIEZO1, further disrupts calcium-dependent transcription, while radiation-induced oxidative injury destabilizes cytoskeletal architecture and amplifies mechanotransduction failure [175,176].

Mitochondria constitute a major convergence node: microgravity alters mitochondrial dynamics and bioenergetics, whereas radiation enhances reactive oxygen species (ROS) generation through ionization-mediated electron-transport-chain disruption. Together, these effects establish a self-reinforcing oxidative cycle that damages nuclear DNA, membrane lipids, and cytoskeletal proteins [173,174], while sustained oxidative stress activates p53-mediated DNA damage responses linked to senescence, apoptosis, inflammatory remodeling, and long-term genomic instability.

Proteostasis provides an additional adaptive network integrating mechanical stress, oxidative injury, and cellular survival. Heat shock proteins (HSPs), particularly HSP70 and HSP27, preserve protein-folding integrity, stabilize cytoskeletal structures, and suppress apoptosis under combined mechanical and oxidative stress [177]. HSP-mediated stabilization supports endothelial vascular integrity and inflammatory regulation, whereas prolonged skeletal-muscle unloading reduces HSP expression and proteostatic responsiveness [177,178,179]. Immune-cell HSP dynamics are similarly sensitive to combined radiation and mechanical stress, highlighting proteostasis as a central regulator of spaceflight adaptation [180].

Failure of these interconnected systems drives cellular reprogramming characterized by impaired proliferation and differentiation, apoptosis, metabolic remodeling, and senescence-associated secretory phenotype (SASP) signaling. In skeletal tissues, mesenchymal stem cells and osteoblasts show suppressed osteogenic differentiation and dysregulated RUNX2 signaling, directly linking mechanotransduction failure to impaired bone formation [181]. These changes propagate to bone loss, muscle atrophy, endothelial dysfunction, immune dysregulation, and systemic metabolic remodeling. Cephalad fluid shifts further redistribute hemodynamic forces, reinforcing skeletal unloading and cardiovascular deconditioning while disrupting inter-organ communication among bone, muscle, adipose, and immune systems [176,182].

Collectively, evidence supports a convergent systems model in which microgravity-induced mechanotransduction failure and radiation-induced genomic instability synergistically drive multisystem degeneration. Their convergence on cytoskeletal integrity, mitochondrial function, redox balance, DNA damage signaling, and proteostasis produces dysfunction beyond additive stress effects. Notably, many spaceflight phenotypes resemble accelerated aging hallmarks, including mitochondrial dysfunction, stem-cell exhaustion, chronic oxidative stress, impaired proteostasis, and cellular senescence, suggesting that spaceflight may represent a compressed model of systemic aging biology.

Major gaps remain in chronic low-dose radiation effects, long-duration adaptive remodeling, and the interactive biology of combined stressors under deep-space conditions. Existing models insufficiently integrate mechanotransduction, mitochondrial signaling, DNA damage responses, and proteostasis into unified predictive frameworks. Future progress in space biomedicine will require systems-level integration of multi-omics profiling, computational modeling, and longitudinal experimental validation to develop predictive countermeasures for long-duration human space exploration [173,174].

## 9. Future Perspectives and Translational Challenges

### 9.1. Future Perspectives and Emerging Technologies

Space biomedicine is shifting from descriptive studies toward predictive, mechanistically grounded models of physiological adaptation to extreme environments, supported by systems biology, computational modeling, and miniaturized platforms that enable multi-scale characterization of responses to microgravity and radiation [1,58,118].

Emerging approaches, including organ-on-chip models, space omics, CRISPR-based functional genomics, organoids, digital twins, and AI, may improve mechanistic, causal, and predictive resolution, but their translational utility requires validation under authentic spaceflight conditions. Early studies demonstrate applications across biological scales, including sarcopenia-like transcriptional remodeling in muscle constructs, mitochondrial dysfunction in cardiac tissues, functional interrogation of stress-response, immune, and mitochondrial networks, and targeted genome editing on the International Space Station [183,184,185,186,187,188,189,190]. Space omics remains largely correlative [3,5,186], whereas integration with functional genomics, organoid models, and computational approaches could help resolve gravity-sensitive regulatory circuits; AI-driven digital twins and NASA GeneLab analyses may further support individualized trajectory modeling and simulation-based space medicine [191,192,193].

Microgravity may also serve as a unique perturbation system for investigating disease and regeneration mechanisms [194]. Space-based protein crystallization could enhance structural resolution for drug discovery [195], while orbital biomanufacturing may generate 3D tissue constructs and stem cell-derived organoids with altered developmental trajectories, providing insight into gravity-dependent growth, differentiation, and aging [196,197,198]. In Saudi Arabia, the Genomic Medicine Center of Excellence at King Faisal Specialist Hospital and Research Centre supports multi-omics and precision-medicine research [59], the Saudi Human Genome Program provides population-scale genomic infrastructure [39], and the Saudi Space Agency’s BioGravity initiative extends this ecosystem toward microgravity research and international collaboration. Aligned with Vision 2030 and the National Biotechnology Strategy 2040, these initiatives may contribute to Saudi Arabia’s emerging role in precision space health [39,59,60].

Major unmet needs include limited long-duration human datasets, methodological heterogeneity, incomplete fidelity of Earth-based analogs, and insufficient characterization of combined space stressors. Progress will require standardized experimental frameworks, improved autonomous orbital platforms, validated cross-scale models, longitudinal human studies, causal rather than correlative validation, and systematic characterization of microgravity–radiation interactions and inter-individual variability. Emerging technologies may facilitate these priorities, but their predictive and translational value requires rigorous validation before application to long-duration human spaceflight.

### 9.2. Translational and Regulatory Challenges

Progress in space biology remains constrained by limited sustained exposure to authentic microgravity, restricting reproducibility and mechanistic resolution across molecular and physiological scales [47,176]. Orbital missions, parabolic flights, clinostats, and random positioning systems only partially reproduce the spaceflight environment; residual sedimentation, convection, and fluid shear can generate mechanical artifacts that perturb mechanotransductive signaling and cytoskeletal organization [176]. Distinguishing intrinsic gravity-dependent responses from platform-induced effects therefore remains a central challenge, while short experimental durations limit characterization of long-term adaptive remodeling and chronic physiological responses [176].

Mechanistic interpretation is further complicated by incompletely resolved fluid–cell interactions under altered gravity. Interactions among fluid dynamics, cellular rheology, and density-dependent sedimentation introduce uncertainty into force transmission and intracellular mechanobiology [176]. Restricted orbital accessibility, low throughput, and high operational costs further constrain statistical robustness and accurate predictive modeling of microgravity adaptation [176,199].

Concurrent space radiation adds another major confounder. Ionizing radiation induces DNA strand breaks, oxidative stress, mitochondrial dysfunction, chromosomal instability, and epigenetic dysregulation [173,174], while microgravity and radiation converge on oxidative, inflammatory, mitochondrial, and transcriptional pathways [47]. This overlap complicates causal attribution because similar molecular phenotypes may arise from distinct stressors. High-linear energy transfer particles, particularly high atomic number and energy (HZE) ions, may intensify these effects through persistent clustered DNA lesions that are inefficiently repaired and highly cytotoxic [173,174].

Translational progress is also limited by infrastructural and regulatory fragmentation. Dependence on governmental and commercial launch systems, limited payload capacity, restricted automation, and constrained experimental flexibility reduce acquisition of high-resolution longitudinal datasets required for systems-level analyses [47]. The absence of harmonized international governance frameworks contributes to heterogeneous biosafety standards, inconsistent validation pipelines, and fragmented data-sharing infrastructures, weakening reproducibility and interoperability and complicating integration of multidimensional biological datasets [47,200]. Ethical and jurisdictional uncertainties regarding ownership and downstream use of extraterrestrial biological materials may further affect translational implementation and public trust [47].

Addressing these barriers will require coordinated validation across experimental platforms, standardized protocols and reporting frameworks, improved longitudinal human data, and harmonized regulatory and data-sharing structures. Emerging experimental and computational approaches may support this transition, but their predictive and clinical utility remains to be established. Ultimately, translation will depend on convergence among mechanobiology, radiation biology, systems science, and harmonized regulatory frameworks to establish reproducible, predictive, and clinically relevant space biomedicine [47,174].

## 10. Conclusions

The evidence does not support treating “microgravity” as a single biological exposure. Instead, biological responses arise from the interaction of gravitational unloading with radiation, fluid dynamics, confinement, circadian disruption, and experimental platform effects. Across omics studies, a recurring convergence is observed around mechanotransduction, mitochondrial/redox dysfunction, DNA damage responses, immune regulation, and host–microbiome interactions; however, most human spaceflight evidence remains associative and model-dependent. Therefore, the major research priority is not simply generating more omics data, but establishing causal, longitudinal, cross-platform models that distinguish genuine gravity-dependent mechanisms from stressor- and platform-specific effects.

## Figures and Tables

**Table 1:** Saudi genomic research infrastructure and its relevance to space bioscience [30,31,34,35,38,40,41].

Infrastructure/Initiative	Primary Terrestrial Objectives	Relevance to Space Bioscience and Microgravity Research
Saudi Human Genome Program (SHGP)	Sequence 100,000 genomes, identify pathogenic variants, and reduce rare genetic disease through targeted screening.	Supplies the population-specific variation baseline needed to identify susceptibility to spaceflight stressors such as radiation and microgravity.
Saudi Pangenome Project	Build an accurate, diverse genomic reference covering Saudi Arabia’s major regions.	Sharpens multi-omics work in space biology, improving detection of structural variants and epigenetic change during missions.
KFSHRC Genomics Medicine Centre	Run clinical genomics, precision diagnostics, and translational research for inherited disorders.	Provides advanced genomic and transcriptomic analytical capabilities relevant to microgravity research, using next-generation sequencing to study microgravity-induced transcriptomic change.
Biogravity Initiative (SSA)	Equip scientists to conduct microgravity research and strengthen the Kingdom’s standing in space science.	The coordinating layer connecting genomic researchers to spaceflight opportunities for cellular experiments and drug discovery.
National screening programs	Block transmission of the severe autosomal recessive disorders made common by high consanguinity.	May provide population-scale genotype and phenotype datasets useful for comparative analyses of biological responses to extreme physiological stress.

**Table 3:** Major Milestones in Genomic and Biomedical Research under Microgravity. The table highlights major space missions and their scientific contributions. It outlines the progression from the establishment of long-term experimental platforms aboard the International Space Station to advanced multi-omics human studies, model organism research, microbial and plant investigations, and recent international and regional missions. Collectively, these milestones have enhanced understanding of molecular and physiological responses to spaceflight and are contributing to the development of precision medicine and translational biomedical applications [1,3,4,5,53,56].

Year	Mission/Program	Milestone	Key Scientific Contribution
2000	International Space Station (ISS)	Continuous human presence in microgravity begins	Enabled long-term biological and biomedical experiments in space
2015	NASA Twins Study	First integrated human multi-omics study in space	Identified genomic, epigenomic, and immune changes during long-duration flight
2016	Rodent Research Missions (NASA)	Mammalian model studies in microgravity	Revealed muscle atrophy, bone loss, and gene expression changes
2018	Microbial & Plant Omics Experiments (ISS)	Omics studies on microbes and plants	Demonstrated altered gene expression and stress adaptation pathways
2021	Inspiration4 Civilian Mission	Expansion of human spaceflight research	Included biomedical sampling and health monitoring in civilians
2023	Axiom Mission 2 (Saudi participation)	Regional contribution to microgravity science	Conducted experiments on human health, cell biology, and space medicine
2024	Space Omics and Medical Atlas (SOMA)	Largest integrated space omics database	Enabled cross-mission genomic and biomedical data integration

**Table 4:** Microbial and Microbiome Alterations under Microgravity and Their Biomedical Implications [3,77,78,79].

Compartment	Observed Alteration	Mechanistic Driver	Functional Consequence
Skin	Decreased Gammaproteobacteria and Betaproteobacteria; expansion of opportunistic genera	Environmental stress; immune modulation	Impaired pathogen exclusion; barrier dysfunction; increased infection susceptibility
Nasal	Increased Staphylococcus, Corynebacterium, and Bifidobacterium	Habitat microbial exchange; confinement ecology	Upper airway colonization; respiratory inflammation
Gut—Compositional	Increased Firmicutes/Bacteroidetes ratio; decreased Clostridiales populations	Metabolic stress; dietary and environmental shifts	Reduced SCFA production; intestinal permeability
Gut—Metabolic	Altered SCFA and tryptophan metabolite profiles	Host–microbial metabolic dysregulation	Cytokine imbalance; mucosal immune disruption
Multi-site	Coupled microbial and immune remodeling	Systemic biological adaptation to spaceflight	Dynamic host–microbiome–immune coupling

**Table 5:** Ethical and legal considerations in genome-editing technologies and their relevance to Saudi microgravity research [103,104].

Technology	Cell Type/Inheritance	Main Ethical Concern	Islamic Bioethics Lens	Relevance to Space Research
CRISPR-Cas9 germline editing	Heritable	Consent of future generations, safety, eugenics risk	Therapeutic use may be discussed; enhancement is strongly discouraged	Could arise in future embryo or gamete editing for space adaptation
Base editing	Somatic or germline	Unintended heritable effects, long-term safety	Subject to the same maqāṣid-based scrutiny as other editing tools	Relevant to precision correction of disease variants
Prime editing	Somatic or germline	Novelty, governance gap, safety validation	Permissible only if therapeutic, safe, and necessary	Potential for highly precise disease correction
Mitochondrial replacement therapy	Reproductive/lineage-related	Parentage, heredity, identity, donor contribution	Sensitive because of lineage and reproductive implications	May become relevant in future reproductive planning for space missions
In vitro gametogenesis/embryo editing	Heritable	Embryo status, consent, future child welfare	Highly restricted; major fiqh concerns	Could emerge in long-duration settlement scenarios
Somatic editing for astronaut health	Non-heritable	Safety, fairness, enhancement boundary	More defensible if therapeutic and necessary	May be used for radiation injury or disease prevention

**Table 6:** Comparative overview of advanced experimental models used in microgravity research.

Platform	Key Strengths	Main Limitations	Typical Applications
Organoids and spheroids	Self-organizing 3D architecture; tissue-specific cell–cell and ECM interactions; modeling of developmental and pathological processes	Incomplete cellular heterogeneity; lack of vascularization; line-to-line variability; limited long-term maturation	Neurodegeneration, cardiovascular dysfunction, radiation injury, stem cell behavior, tumor biology
Organ-on-a-chip/MPS	Controlled fluid flow and mechanical cues; multi-tissue integration; compact and automatable for spaceflight	Simplified cellular arrangements; limited tissue maturation; technical complexity of multi-organ coupling	Drug screening, toxicity testing, organ–organ communication, ISS-adapted experiments
Hybrid bioprinted MPS	Combines biofabricated 3D tissue architecture with dynamic perfusion; supports vascularization and tumor–vascular interactions	Fabrication complexity; need for specialized bioinks and printers; reproducibility across laboratories	Vascularized tumor models, regenerative tissue constructs, mechanobiology studies
Ground-based simulators	Accessible, reproducible, and cost-effective; suitable for hypothesis generation and screening	Residual acceleration, vibration, and shear stress; no radiation or true unloading; partial physiological fidelity	Mechanotransduction, microbial adaptation, musculoskeletal and stem cell studies
Orbital and beyond-LEO platforms	True microgravity, radiation, and combined stressors; long-duration experiments	Limited access; high cost; constrained payload, crew, and return logistics	Validation studies, long-term tissue engineering, astrobiology, deep-space health research

## Data Availability

No new data were created or analyzed in this study. Data sharing is not applicable to this article.

## Abbreviations

The following abbreviations are used in this manuscript:

AI	Artificial Intelligence
Ax-2	Axiom Mission 2
CRISPR	Clustered Regularly Interspaced Short Palindromic Repeats
ESA	European Space Agency
GC-MS	Gas Chromatography–Mass Spectrometry
ISS	International Space Station
KACST	King Abdulaziz City for Science and Technology
KFSHRC	King Faisal Specialist Hospital and Research Centre
KAUST	King Abdullah University of Science and Technology
KFUPM	King Fahd University of Petroleum and Minerals
LC-MS	Liquid Chromatography–Mass Spectrometry
MRT	Mitochondrial Replacement Therapy
NASA	National Aeronautics and Space Administration
RNA-seq	RNA Sequencing
ROS	Reactive Oxygen Species
SCFA	Short-Chain Fatty Acids
scRNA-seq	Single-Cell RNA Sequencing
SFDA	Saudi Food and Drug Authority
SHGP	Saudi Human Genome Program
SOMA	Space Omics and Medical Atlas
SSA	Saudi Space Agency
T2T	Telomere-to-Telomere
UNOOSA	United Nations Office for Outer Space Affairs
WGS	Whole Genome Sequencing
